# The Impact of Prior Beliefs about Volatility on Adaptive Behavior

**DOI:** 10.5334/joc.504

**Published:** 2026-05-27

**Authors:** Anna Bleser, Gereon R. Fink, Simone Vossel, Paola Mengotti

**Affiliations:** 1 Cognitive Neuroscience, Institute of Neuroscience & Medicine (INM-3), Forschungszentrum Jülich, 52425 Jülich, Germany; 2 Department of Psychology, Faculty of Human Sciences, University of Cologne, 50923 Cologne, Germany; 3 Department of Neurology, Faculty of Medicine and University Hospital Cologne, University of Cologne, 50937 Cologne, Germany

**Keywords:** learning rate, Rescorla-Wagner model, false prior belief, probabilistic reversal-learning, reaction times, predictive coding

## Abstract

Humans adapt to environmental changes by balancing empirical observations with prior beliefs and evaluating if unexpected events indicate a true change. The specific factors that govern updating behavior in dynamic environments remain to be elucidated. We here examined how prior beliefs about environmental volatility affect updating of cue-target contingencies, particularly when observations violate these beliefs. Thirty-two participants completed two versions of a probabilistic reversal-learning task, in which auditory cues signaled the location of a subsequent visual target stimulus. In a reactive task version, participants indicated the target location after its appearance; in a predictive task version, they predicted the target’s location based on the cue information. Cue-target contingencies either remained stable or reversed once within a block, thereby creating a stable and a reversal environment. Before each block, participants received either true or false information about volatility, i.e., about whether the cue-target contingency would remain stable or change. We analyzed reaction times (reactive task) and choices (predictive task) with model-free measures and a Rescorla-Wagner learning model. Participants generally adapted to the contingency changes in both tasks. In the reactive task, prior beliefs had no significant effect. In the predictive task, believing that a reversal environment was stable reduced learning rates. In stable environments, falsely believing the environment contained a reversal increased decision noise, reduced accuracy and increased choice variability. These findings demonstrate that prior beliefs about volatility shape updating in response to task demands and environmental structure.

## Introduction

Human cognition is deeply rooted in the brain’s capacity to predict and adapt to environmental changes through empirical observation. These predictions rely on internal models of the world that are shaped by past experiences and guide perception, decision-making, and action. When predictions are violated, this discrepancy is resolved by updating the internal models of the world, a process central to the framework of predictive coding (Friston, 2010). In these frameworks, predictions are often described as ‘beliefs’ in a functional/mechanistic rather than mentalistic sense (Behrens et al., 2007; Topel et al., 2023). Updating is particularly relevant when the current internal model relies on a false prior belief (e.g., one that has become outdated or was based on unreliable/misleading information).

Current evidence on how false priors affect belief updating presents a mixed picture. Mengotti et al. (2017) demonstrated that false prior beliefs about cue-target contingencies led to faster belief updating of the actual contingency (i.e., higher learning rates). However, other work has suggested that some prior beliefs may persist even when contradicted by sensory evidence (Yon et al., 2023). While the precise mechanisms remain unclear, it is evident that false priors may have a substantial impact on how beliefs are updated.

Another factor that influences belief updating is how stable or volatile the world is perceived to be. The world is inherently dynamic, with contingencies often shifting unpredictably over time. In such volatile environments, a key challenge is to decide whether an unpredicted event signals a true environmental change, or whether it should be treated as an odd-one out event (noise) that does not require an update of the internal model. This involves a delicate balance of updating behavior, to avoid both insufficient updating (leading to too rigid, outdated beliefs), and excessive updating (overreacting to noise). Influential formal models of prediction updating propose that beliefs about the volatility of the environment are used to navigate this uncertainty: When an environment is believed to be stable, the brain prioritizes prior beliefs over incoming sensory data, thereby reducing updating. Conversely, when an environment is believed to be more dynamic, greater weight is assigned to incoming evidence, leading to faster updating (Behrens et al., 2007; Mathys et al., 2011; Piray & Daw, 2020).

Computational studies have already examined how inferred higher-order beliefs about environmental volatility modulate updating, typically by manipulating the environmental volatility directly (e.g., Iglesias et al., 2013; Lawson et al., 2017). However, the effects of *false prior beliefs* about volatility remain unclear. False priors about volatility may bias the initial belief state and influence how sensory evidence is weighted during updating, thereby inducing higher-order uncertainty, i.e., uncertainty about environmental volatility (Sandhu et al., 2023). This line of research is particularly relevant, since distortions of higher-order beliefs (i.e., misestimations of volatility) are assumed to play a critical role in neuropsychiatric disorders (Hauke et al., 2024; Lawson et al., 2017; Pulcu & Browning, 2019).

These distortions seem to manifest differently across different clinical conditions (for review, see Sandhu et al., 2023). For instance, anxiety has been linked to increased uncertainty about volatility (Hein et al., 2021), individuals with autism show faster updating of higher-order beliefs about volatility (Lawson et al., 2017), and individuals with paranoia exhibit a strong belief in high volatility that persists despite contrary evidence (Reed et al., 2020). By inducing different prior volatility beliefs within the same healthy individuals, the present study aimed to characterize the basic mechanisms of volatility-dependent belief updating that navigate uncertainty, using behavioral and computational signatures.

Previous work has begun to characterize the impact of higher-order prior beliefs about volatility on updating in healthy volunteers. In a previous study by Schiffer et al. (2017), participants received explicit probabilistic instructions about environmental volatility before performing a reversal-learning task. Volatility instructions led to faster adaptations to unannounced rule switches and modulated the stimulus-preceding negativity in the EEG. However, participants in that study were fully and accurately informed about the instruction-environment contingencies, warranting further investigations into the effects of false volatility priors. More recently, Jedlovszky, Corlett & Yon (2024) investigated how expecting more or less volatility influences behavior in a reward learning task with multiple contingency changes. They found that expecting greater volatility led to higher decision noise and increased switching behavior, without affecting learning rates.

The present study extends this line of research by directly manipulating the veridicality of higher-order volatility beliefs. Moreover, beyond these higher-order beliefs about environmental volatility, belief updating may also depend on task demands, such as the degree of active action required within the environment. Actively interacting with an environment leads to slower updating than passive observation (Weiss et al. 2021; see also Yon et al., 2023 for a similar notion). Here, we tested whether explicit prediction demands alone are sufficient to induce a similar stabilization of belief updating. We therefore manipulated volatility beliefs in two different tasks. In a predictive task, participants were required to make an explicit prediction of the target location by pressing a button before the target appeared. In contrast, in the reactive task, participants responded after target appearance and could therefore more passively observe the target location, relying on simple stimulus-driven detection. This allowed us to examine how task demands (in particular the more or less explicit and controlled processing of predictions) modulate the volatility prior effects on learning, by comparing belief updating under similar perceptual but distinct response-related processes. Past literature characterizing belief updating processes in volatile environments used either predictive (Behrens et al., 2007, Iglesias et al., 2013) or reactive tasks (Lawson et al., 2017, Vossel et al., 2014), and, to the best of our knowledge, a systematic comparison of these tasks is missing.

To this end, we combined a reversal learning with a Posner spatial cueing task. The Posner task (Posner, 1980) uses cues (e.g., central arrows) to provide advance information about the location of a target stimulus, thereby guiding attention and speeding up responses to the target when the cue is valid in the majority of trials. The difference in response times to validly and invalidly cued targets is affected by the specific cue-target contingency (i.e., the proportion of valid and invalid trials), even when this contingency changes unpredictably during the task, creating a volatile environment (Vossel et al., 2014). Reversal learning tasks require participants to learn and adapt to reversals in cue-outcome or cue-response contingencies, with participants differing in the degree of persistence they show before discovering the reversal of the contingency (for a review, see Izquierdo et al., 2017). In the present study, we combined these two paradigms so that the probability of a cue indicating a specific target location was inverted (i.e., reversed) in some experimental blocks. In two task versions with different response requirements (predictive versus reactive, see above), cue-target contingencies remained either stable or reversed once per experimental block. Importantly, participants received either true or false prior information about the occurrence of these contingency changes in different blocks. Blocks that include a contingency change can be considered to be more volatile when compared to blocks with stable cue-target contingencies.

To quantify belief updating, we combined conventional model-free analyses of reaction times (RTs) and accuracy rates (reactive task), and choice probabilities (predictive task) with a model-based approach. Unlike model-free measures, which capture only observable behavioral patterns (usually averaged across multiple trials), model-based analyses estimate the underlying belief trajectories that drive these patterns at the trial-by-trial level. Applying models such as the Rescorla-Wagner (RW) learning model (Rescorla & Wagner, 1972) to trial-wise responses allows the derivation of condition- and participant-specific learning rates that reflect how quickly participants update beliefs about stimulus (e.g., cue-target) associations after new observations. The RW model describes belief updating as a trial-by-trial adjustment driven by prediction errors, which are weighted by the learning rate parameter, such that higher learning rates lead to faster updating. This simple, widely used framework has already been successfully applied to behavioral data in similar tasks (Jedlovszky et al., 2024; Mengotti et al., 2017).

Here, we hypothesized that updating behavior (learning rates) would be significantly affected by prior volatility information. Since belief updating is faster when the environment is perceived as more volatile (Behrens et al., 2007), we expected opposite modulations of belief updating for false priors in stable versus reversal environments: While a false prior should lead to faster updating (more substantial behavioral changes and higher learning rates) than a true prior in stable blocks (by signaling a reversal environment), it should lead to slower updating in reversal blocks (by signaling a stable environment). Since one study showed that prior volatility beliefs may affect response parameters such as decision noise rather than learning rates (Jedlovszky et al., 2024), these parameters were analyzed exploratorily. As for task-related differences, since more explicit prediction processing could result in higher perceived environmental stability (Weiss et al., 2021), we hypothesized that updating would generally be slower in the predictive task.

## Methods

### Participants

We ran an a priori power analysis based on effect sizes reported in the TMS studies by Mengotti et al. (2017, 2022), in which a paradigm with true and false priors about cue-target contingencies was employed and a Rescorla-Wagner model was used to derive learning rates. Using the effect sizes of the paired t-test on the learning rate between true and false priors for the sham coil condition (Mengotti et al., 2017: Cohen’s d = 1.5; Mengotti et al., 2022: Cohen’s d = 0.65) yielded a suggested sample size of 7 to 27 participants, respectively (alpha = 0.05, power = 0.9; G∗Power Version 3.1.9.5). Based on the more conservative sample size estimate of at least 27 participants, a total of 38 participants were initially recruited for this study to account for possible exclusions. All participants were right-handed, healthy, and had normal or corrected-to-normal vision. Six participants were excluded for the following reasons: three for failing to meet the accuracy inclusion criterion in the reactive task (accuracy within two standard deviations (SDs) of the group’s mean), two for incomplete data, and one for ambidexterity. Thus, the final sample consisted of 32 participants (18 male, 14 female, age range: 19–38 years, mean age: 27 years). The study was approved by the ethics committee of the German Society of Psychology (DGPs) and was performed following the Code of Ethics of the World Medical Association (Declaration of Helsinki). Participants provided written informed consent before their inclusion in the study and were informed about their right to withdraw at any time without penalty. They received monetary compensation for their time. This study was not preregistered.

### Experimental design

**Figure 1:**
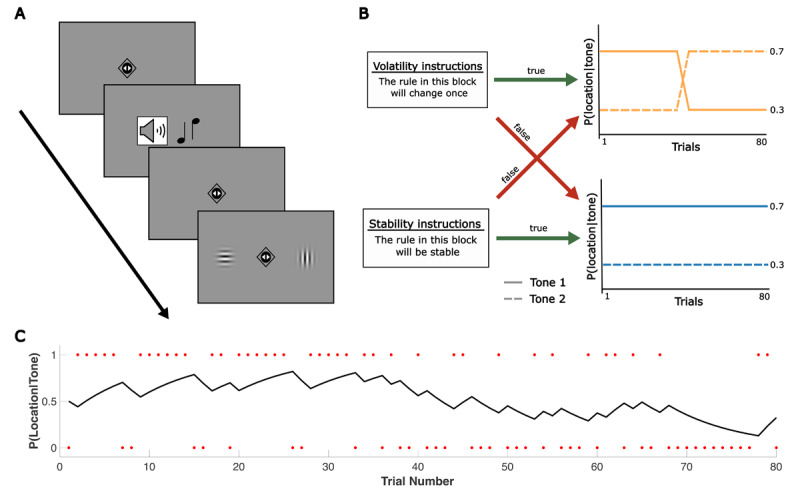
Overview of experimental setup and modelling approach. **(A)** General trial sequence for both tasks. Whereas two triangles were shown inside the fixation diamond in the predictive task, two small circles were depicted at fixation in the reactive task to avoid confusion of the two different response requirements. A high or low tone predicted the target’s left or right location with a certain probability. Participants were asked to indicate the target’s location after its appearance (reactive task) or to predict the target’s location before it appeared (predictive task). **(B)** Overview of the experimental conditions. Participants completed four blocks per task. Blocks were either reversal or stable blocks (i.e., involved a reversal of cue-target contingencies or not). At the beginning of each block, true or false instructions about the volatility of the block were presented. **(C)** Example of the output from the learning model, showing a participant’s estimated probability of tone 1 leading to target location 1 across trials of a reversal block. Red dots indicate the trial-wise outcome *u_t_*, coded relative to the cue-target contingency in the first half of the block: *u_t_* = 1 if the outcome matched this initial contingency, and *u_t_* = 0 if the outcome did not match the initial contingency. Further examples of trajectories per block type are provided in Supplementary Figure S1.

In a within-subject cross-over design, participants sequentially completed the two versions of the location-cueing/reversal learning task (see Figure 1A). All stimuli were generated and displayed on a gray background using PsychoPy (2023.2.3) on a Windows laptop with 1920 × 1080 pixels resolution at 60 Hz refresh rate with a viewing distance of 60 cm. Throughout the whole experiment, participants were instructed to fixate on the central diamond presented at the center of the screen. At the beginning of a trial, participants heard a 300 ms auditory cue. This cue could be a high tone (587.33 Hz) or a low tone (349.23 Hz), predicting either a left or right location of a subsequent visual target. To ensure that participants were able to differentiate between the two tones, a 5 min sound test was performed before the start of the experiment. After an interval of 500 ms (reactive task) or 1350 ms (predictive task) after the cue, a horizontally and a vertically rotated Gabor grating (4° × 4°, spatial frequency: 1.5) were presented for a duration of 350 ms to the left and right of the fixation point (with 6° eccentricity). One of the two gratings (horizontal or vertical) was defined as the target stimulus. In the reactive task, participants were asked to indicate the target location with a button press using the index or middle finger of their right hand within a 1000ms response window after the target had been presented. In the predictive task, participants were asked to use the button press to predict the target location based on the cue before the target’s appearance. The intertrial interval varied between 1300 ms and 3000 ms in both tasks. The target appeared on the left and right sides with equal probability in each task. The order of task versions, the target assignment (horizontal or vertical), as well as the tone-target location mapping, was counterbalanced across participants.

In each task version, participants completed four blocks, each comprising 80 trials. The order of the four blocks was pseudorandomized across participants. However, to ensure comparability between the two task versions, the same sequence of blocks and trials was used for a given participant. Blocks were either stable or reversal blocks. In stable blocks, the cue-target contingency remained *stable* at a probability of 0.7 (e.g., a high tone predicted a right target in 70% of trials and a left target in 30% of trials). In reversal blocks, the cue-target contingency *reversed* after half of the trials (see Figure 1B). Note that reversal blocks were the more volatile block type in our design, relative to stable blocks, but (unlike many existing studies on volatility) contained only one reversal. Participants were not explicitly informed about these contingencies. However, consistent with previous studies (Iglesias et al., 2013), they were told that the relationship between the auditory cue (ht = high tone, lt = low tone) and target location (r = right target, l = left target) followed a structured pattern (‘rule’) with:


$$P\left(r|ht\right)=1-P\left(l|ht\right)\;=P\left(l|lt\right)=1-P\left(r|lt\right)$$

To manipulate prior beliefs about environmental volatility, each block was combined with true or false prior information about changes in cue-target contingency (see Figure 1B). In half of the blocks, participants received the instruction: ‘The rule in this block will stay the same’ (stability prior); in the other half, the instruction was: ‘The rule in this block will change once’ (reversal prior). This information was presented on the screen for 4 s at the beginning of each block. Additionally, there was a small reminder of this instruction (either ‘stable’ or ‘change’) at the top of the screen for the whole duration of a block. Each block represented one of the four conditions (stable block/true prior (i.e., stability prior), stable block/false prior (i.e., reversal prior), reversal block/true prior (i.e., reversal prior), reversal block/false prior (i.e., stability prior)). Although participants were informed that the prior information could be false, they did not know how many blocks were false. To ensure that participants attended to this prior information, they were asked whether it was correct or incorrect at the end of each block.

For each condition, a different trial sequence was created and kept constant throughout the experiment to prevent participants from learning and anticipating the sequences within environments. To ensure comparability of priors within environments, we avoided repeating sets of three unexpected trials and ensured that each block phase within environments contained a similar number of unexpected trials (stable/true: 9,6,9; stable/false: 8,8,8; reversal/true: 8,13,19; reversal/false 8,13,19). The total duration of both task versions was 46 minutes.

Before each task, participants underwent a short training session consisting of four blocks. The first two blocks each contained 30 trials. Both blocks had a stable cue-target contingency (P(target location|tone) = 0.7), allowing participants to familiarize themselves with the main task’s contingency level. The third and fourth blocks comprised 60 trials each and were presented consecutively, with one block containing a contingency reversal and the other remaining stable. Participants were instructed to identify which block included the reversal.

### Data analysis

#### Model-free analysis

Preprocessing of behavioral data was performed with Python in Visual Studio Code (Microsoft Corporation, n.d.), and statistical analyses were conducted with Jasp (Version 0.19). The model-free group-level analysis employed repeated-measures analyses of variances (ANOVAs) with Greenhouse-Geisser correction when sphericity was violated. Accuracy of responses to the question trials after each block in each task was analyzed using a 2 × 2 repeated-measures ANOVA with factors *environment* (stable, reversal) and *prior* (true, false).

In the reactive task version, we analyzed reaction times (RTs) and accuracy. RT outliers (i.e., trials with RTs deviating more than two standard deviations from the participant’s mean RT) were excluded from the RT analysis. Cueing effects were calculated based on the initial block-specific cue-target contingency in the first block half for RTs (RT unexpected location – RT expected location) as well as for accuracy (% correct unexpected location – % correct expected location).

In the predictive task, we analyzed both the choices (location predictions) and switches between them. For the binary choice data (left/right predictions), participants’ responses were coded to distinguish between responses matching the initial cue-target contingency and those matching the reversed contingency. Specifically, participants’ choices were coded as one if they matched the most likely outcome according to the initial cue-target contingency in the first block half, and zero if they matched the inverse contingency (i.e., the contingency of the alternative outcome and the contingency after the reversal in reversal blocks). This coding remained constant throughout a block. Choice probabilities were calculated by dividing the sum of responses coded as one by the total number of trials in the relevant condition. For the analyses of switching behavior, a switch was defined as a choice that differed from the choices made in the four trials immediately preceding the trial. This definition was chosen to reduce sensitivity to isolated noisy responses and to better capture sustained behavioral adjustment across several trials. To ensure one count per switch event, consecutive switches were removed. The number of switches was averaged across trials to calculate the switch rate.

To capture different processes related to the initial learning and the updating of the learned cue-target contingencies, the 80 block-wise trials were divided into three sections for both task versions: trials 1–27 (initial learning phase), trials 28–53 (change expectation phase in the middle section, associated with increasing anticipation of a possible rule reversal), and trials 54–80 (adaptation phase). As participants did not know exactly when the reversal occurred, the passage of trials without a change is expected to gradually increase uncertainty about the cue-target contingency, leading to a greater expectation that a reversal may occur. The middle section of the block should therefore represent the period when expectations of a change are relatively high, even before the reversal actually occurs. Therefore, dividing the blocks into three sections allowed us to capture this time of heightened expectations.

At the group level, we first analyzed each task separately. Cueing effects on RTs and accuracy (reactive task) as well as location choice probabilities and switching behavior (predictive task) were analyzed using 3 × 2 × 2 ANOVAs with the factors *block phase* (initial learning, change expectation, adaptation), *environment* (stable, reversal), and *prior* (true, false). Updating of cue-target contingencies after a rule change should be reflected in reversed cueing effects (reactive task) and in reversed (i.e., <0.5) choice probabilities (predictive task) in the last third of reversal blocks, with no changes in stable blocks (i.e., in significant *block phase* x *environment* interactions). Moreover, we expected significant *block phase* x *environment* x *prior* interactions, indicating a modulation of updating behavior by prior volatility information. For the switching data of the predictive task, we expected participants to switch more under volatility instructions (i.e., with true prior information in reversal blocks and with false prior information in stable blocks) (Jedlovszky et al., 2024).

Additionally, to further examine how choice behavior varied as a function of *environment* (stable, reversal) and *prior* (true, false) at a smaller time scale, we conducted a sliding-window analysis in the predictive task. We applied a sliding window with a size of 4 trials and a step size of one trial to the choice data for each participant and condition. Within each window, we calculated the proportion of choices following the initial cue-target contingency. This produced a time series reflecting each participant’s choice behavior across each block. The resulting windowed averages for each participant and condition were subjected to a cluster-based permutation t-test, implemented with the MNE library (Gramfort et al., 2013) to identify time intervals with significant differences between conditions. Clusters were formed based on adjacent time points exceeding a threshold of *p* < 0.05. A total of 10,000 permutations was performed to compute a null distribution. The maximum cluster-level test statistic was compared against this null distribution to control for multiple comparisons, with a significance threshold of p < 0.05 (two-tailed).

In a similar vein, in an exploratory post-hoc analysis of the reactive task, we examined whether belief updating was influenced by the outcome of the previous trial (i.e., whether the previous trial followed the contingency rule or not). We assumed that rule violations in the preceding trial may reveal an effect of prior on RTs. Due to the limited number of trials, this analysis had to be restricted to more frequent/expected trials, i.e., trials where the outcome matched the current cue-target contingency. RTs were analyzed using a 3 × 2 × 2 × 2 ANOVA with factors *block phase* (initial learning, change expectation, adaptation), *environment* (stable, reversal), *prior* (true, false), and *previous trial outcome* (expected, unexpected).

#### Model-based analysis

We performed computational modelling with MATLAB (R2022b, The MathWorks, Inc., MA, USA) and the Hierarchical Gaussian Filter (HGF) functions of the TAPAS toolbox (Frässle et al., 2021). Since the task consisted of short independent blocks, a simple Rescorla-Wagner (RW) model was implemented to capture trial-by-trial updating dynamics, following the procedure in Mengotti et al. (2017, 2022) and Jedlovszky et al. (2024). In this model, the probability of a target location given an auditory cue after observing a trial *t* (*vt* = *P(location*|*tone)*; see Figure 1C for an example) is determined by the following equation:


$$v_{t}=v_{t-1}+\alpha \cdot \delta_{t}$$

Here, the learning rate α determines how strongly prediction errors *δ_t_* (the difference between the observed *ut* and the predicted binary outcome *vt*-1) influence belief updates from trial to trial, with higher values indicating faster belief updating. In each block, the trial-wise outcome *ut* was coded as one if the presented target location was the more likely location according to the cue-target contingency in the first half of a block, and coded as zero otherwise. This binary coding indicates whether participants received an expected or unexpected outcome on each trial, relative to the initial cue-target contingency in each block.

To account for the different response modalities across the two task versions (RTs and binary choices), two distinct response models were applied to map from the participants’ beliefs about the predictive value of the cue to observed responses. For the reactive task, RTs were transformed into response speed (RS = 1/RT) to ensure a more normal distribution. The response model in the reactive task assumed a linear relationship between the RS and the estimated probability that the respective cue will lead to a specific target location before observation of trial ${\hat{v}}_{t}$ = *vt*-1 (Vossel et al., 2014):


$$RS_{t}=u_{t}\left(\zeta_{1}+\zeta_{2}{\hat{v}}_{t}\right)+\left(1-u_{t}\right)\left(\;\zeta_{1}+\zeta_{2}\left(1-{\hat{v}}_{t}\right)\right)\;$$

Here, *ζ*_1_-values represent the intercepts, while *ζ*_2_ determines the slope of the linear function, i.e., the influence of the estimated probability (${\hat{v}}_{t}$) on RS.

For the choice data in the predictive task, a binary softmax response model was applied. The probability of choice/prediction i_t_ = 1 on trial *t* was given by:


$$P\left(i_{t}=1\right)=\;\;\frac{1}{\left(1+\exp\left(-\beta \left({\hat{v}}_{t}-\left(1-{\hat{v}}_{t}\right)\right)\right)\right)\;}$$

where the subject-specific parameter *β* determines the randomness of choices (decision noise). Higher *β* values indicate more deterministic choices, whereas lower values suggest greater response variability (noise). The probability of the alternative choice i_t_ = 0 can be derived by reversing ${\hat{v}}_{t}$ and $\left(1-{\hat{v}}_{t}\right)$ .

The learning rates and response model parameters were fitted separately for each of the four blocks of each task. These values were used for group-level analyses. Complete posterior estimates for all model parameters, as well as prior values used to estimate the model, are provided in Supplementary Tables S2–S4.

##### Model validation and parameter recovery

To assess whether the RW model provided a reasonable account of the data, it was compared to two alternative models. Specifically, the log model evidence (LME) of the RW model was compared to a 3-level HGF model (see Figure S4 for schematic illustration), as well as a 2-level HGF model (Mathys et al., 2011) using random-effects Bayesian model selection (BMS) (Stephan et al., 2009) across all blocks, separately for the two task versions. The BMS indicated that the HGF models had lower model evidence than the RW model across both task versions. The RW model showed a higher exceedance probability (unprotected/protected – reactive task: 1/0.99; predictive task: 0.99/0.87). Complete model comparison methods and results are reported in the Supplementary materials (Parts A-B and Table S1).

In addition, we evaluated the predictive validity of the RW model by comparing observed behavioral responses with responses simulated from the model using the subject-specific parameters. Both observed and simulated responses were grouped according to the model-estimated belief probability ${\hat{v}}_{t}$ into four bins and averaged across subjects. Across both tasks, the observed response patterns closely matched those derived from the learning model under all conditions, supporting the validity of our modelling approach (see Figure S8 of the Supplementary materials).

To assess whether the RW model could capture behavioral patterns observed at the group level, we moreover applied the same model-free behavioral analyses to one simulated dataset generated from the fitted model using each participant’s subject-specific parameter estimates and the same task structure as in the observed data (Palminteri et al., 2017). Overall, the simulated data reproduced all key behavioral effects observed in the empirical data analyses. Detailed statistical results are reported in Supplementary materials (Part C, Figure S5).

In addition, we evaluated the validity of the model fitting procedure using a parameter recovery analysis, separately for the reactive and predictive tasks across each of the four conditions. We generated 20 simulated datasets using distinct random seeds based on the true parameters estimated from the empirical data. Each simulated dataset was fitted with the Rescorla-Wagner model to recover parameter estimates. We calculated Pearson correlation coefficients between the true and recovered parameter values across the 20 simulations for each condition and averaged these correlations (Bağci et al., 2022; Gagne et al., 2020). To quantify effect sizes, we computed Cohen’s ƒ^2^. Values of 0.35 or higher were considered indicative of good parameter recovery (Hauke et al., 2024). Parameter recovery indicated that, in the reactive task, the response model parameters *ζ*_1_- and *ζ*_2_ showed good recovery in all conditions, whereas the perceptual model parameter (learning rate *α*) showed good recovery in all conditions except in the reversal environment with a true prior (for full results see Supplementary materials Table S5; for example, see Figure S6). In the predictive task, all perceptual and response model parameters could be reliably recovered across all conditions (for full results, see Supplementary materials Table S6; for example, see Figure S7).

##### Statistical analysis of model parameters

Due to structural differences between the stable and reversal environments, which can impact the perceptual model parameters, we analyzed the RW model parameters separately for each environment. We used a priori planned paired t-tests to compare learning rates under the different priors for each environment. In addition, we report a 2 × 2 repeated-measures ANOVA on learning rates with the factors *environment* (stable, reversal) and *prior* (true, false) for completeness. We further conducted an exploratory analysis of response model parameters across different priors, specifically the *ζ*_2_-values from the linear response speed model (reactive task) and the *β*-parameter from the softmax decision model (predictive task). Pearson’s correlation coefficient of the *β*-values and the probability of switching were analyzed to relate model-based and model-free measures in each condition.

Finally, to compare the two task versions, ANOVAs on learning rates (the only common parameter across tasks) were computed with factors *task version* (predictive, reactive) and *prior* (true, false), separately for the two environments. To examine the relationship between conditions of the two task versions, Pearson correlation coefficients were analyzed for each pair of corresponding conditions.

All analyses were performed with a significance threshold of p < 0.05. All post-hoc pairwise t-tests conducted to analyze interactions, as well as the correlation analyses, were Bonferroni-corrected for multiple comparisons. For each bar graph presented in the main figures, a corresponding plot showing the underlying data distribution is available in the Supplementary materials (Figure S2).

#### Eye movement data

Eye tracking was used to verify participants’ general compliance with central fixation instructions during the cue-target interval. We recorded eye movements using a Tobii eye-tracking system (X3-120) with a sampling rate of 120 Hz. At the start of the experiment, the system was calibrated and validated to ensure accuracy (validation error around 1° of visual angle). The data were analyzed in Python. The primary focus of the analysis was the interval between the presentation of the cue and the appearance of the target symbol (reactive task: 500 ms, predictive task: 1350 ms), during which fixation was assessed. For each trial, we calculated the percentage of time the participant’s gaze remained within a 1.5° visual angle along the horizontal axis around the fixation during this period, and then averaged these percentages across trials. We also assessed eye movements toward cued versus uncued locations before target onset. For each trial, we computed the proportion of trials with horizontal gaze deviations exceeding 4° (i.e., < –4° on the left, > 4° on the right). The proportions were analyzed using an ANOVA with factors *environment* (stable, reversal), *prior* (true, false), and *side* (cued, uncued) separately for each task. Due to a hardware error that caused incorrect time stamps in the data files, only 10 eye movement datasets were available for analysis. All results of eye-tracking analyses are presented in the Supplementary Materials, Part D.

## Results

### Reactive task

Participants demonstrated high accuracy in the question trials at the end of each block (90 ± 3.13% SEM), suggesting that they attended to the information at the beginning of the blocks and could reliably identify false instructions. This pattern held across all conditions, as the 2 × 2 ANOVA with the factors *environment* and *prior* did not yield any significant results.

For the experimental trials, participants indicated the target location correctly in 95% (±0.3% SEM) of the trials with a mean RT of 500ms (±30 ms SEM).

**Figure 2:**
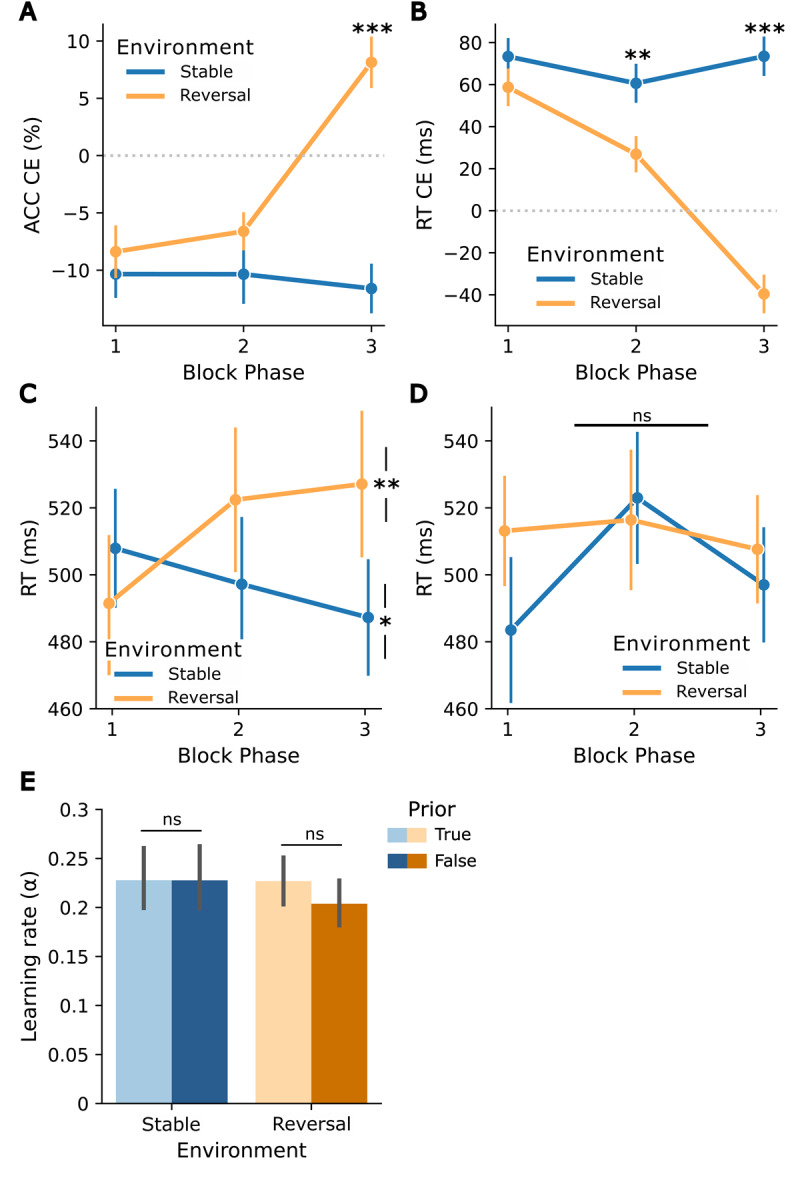
Adaptation to cue-target contingencies and their changes, in relation to prior volatility beliefs in the reactive task. *Block phase* x *environment* interaction for cueing effects (CEs): **(A)** Accuracy CEs (ACC unexpected – ACC expected, based on the contingency in the first half), **(B)** RT CEs (RT unexpected – RT expected, based on the contingency in the first half), both reflecting adaptation to the reversal in cue-target contingency in reversal blocks. **(C)** Post-hoc analysis: more frequent/expected trial RTs after a trial that does not match the cue-target contingency for true priors. Asterisks indicate a difference between the first and last block phases in each environment. **(D)** Post-hoc analysis: more frequent/expected trial RTs after a trial that does not match the cue-target contingency for false priors. **(E)** Learning rate *α* for both environments (stable, reversal) and priors (true, false). Asterisks indicate significance levels: p < 0.05 (*), p < 0.01 (**), p < 0.001 (***). Error bars reflect standard errors of the mean (SEM).

The 3 × 2 × 2 ANOVA conducted on cueing effects in accuracy to examine the effects of *block phase, environment*, and *prior* revealed a significant main effect of *block phase* (*F*(2,62) = 8.05, *p* < 0.001, $\eta_{p}^{2}=0.21$), with post-hoc pairwise t-tests indicating higher accuracy cueing effects in the last phase compared to the first (*t*(31) = 3.4, *p* = 0.006) and second (*t*(31) = 3.2, *p* = 0.009) phase, and a main effect of *environment*, reflecting higher accuracy cueing effects in the reversal environment (*F*(1,31) = 23.07, *p* < 0.001, $\eta_{p}^{2}=0.43$). Additionally, the interaction of *block phase* and *environment* was significant (*F*(1.5,46.45) = 16.66, *p* < 0.001, $\eta_{p}^{2}=0.35$). Post-hoc pairwise t-tests revealed the expected pattern with differences between stable and reversal environments emerging in the last phase of a block (*t*(31) = –5.46, *p* < 0.001). More specifically, the direction of the cueing effects reversed in reversal blocks (when contingencies reversed), but not in stable blocks in the third phase – reflecting adaptation to block-specific contingencies (see Figure 2A). There was no significant *block phase* x *environment* x *prior* interaction (*p* = 0.61) nor an *environment* x *prior* interaction (*p* = 0.13), indicating that the prior did not impact the updating of cue-target contingencies.

The analysis on the cueing effects in RTs using the equivalent 3 × 2 × 2 ANOVA with factors *block phase, environment*, and *prior* yielded a significant main effect of *block phase* (*F*(2,62) = 16.1, *p* < 0.001, $\eta_{p}^{2}=0.34$), with decreasing RT cueing effects over the three block phases (all pairwise t-tests *p* < 0.006), and a main effect of *environment*, reflecting higher RT cueing effects in the stable environment (*F*(1,31) = 48.3, *p* < 0.001, $\eta_{p}^{2}=0.61$). Consistent with the results on the accuracy cueing effects, we observed a significant *block phase* x *environment* interaction (*F*(2,62) = 17.1, *p* < 0.001, $\eta_{p}^{2}=0.36$). Post-hoc pairwise t-tests revealed the expected pattern, with differences in environments emerging in the second (*t*(31) = 3.77, *p* = 0.005) and third phase (*t*(31) = 5.91, *p* < 0.001) of the blocks. More specifically, whereas the stable environment was characterized by stable positive cueing effects, the reversal environment showed reversed (negative) cueing effects in the last phase. (i.e., an adaptation to reversed contingencies; see Figure 2B). In addition, the *block phase* x *prior* interaction was significant (*F*(2,62) = 6.38, *p* = 0.003, $\eta_{p}^{2}=0.17$), reflecting differences in RT cueing effects across the first phase, with larger positive cueing effects for false priors. Similar to the results for the accuracy cueing effects, no significant *block phase* x *environment* x *prior* interaction (*p* = 0.3) and no significant *environment* x *prior* interaction (*p* = 0.32) were observed, indicating that the prior did not affect the updating of cue-target contingencies.

In a post-hoc exploratory analysis, we investigated whether the outcome of the previous trial (i.e., whether the previous trial followed the contingency rule or not) influenced the effect of the prior. The post-hoc 3 × 2 × 2 × 2 ANOVA on expected trial RTs with factors *block phase, environment, prior*, and *previous trial outcome* revealed significant main effects of *block phase* (*F*(2,62) = 4.87, *p* = 0.01, $\eta_{p}^{2}=0.14$), with post-hoc pairwise t-tests indicating increased RTs in the second block phase compared to the first block phase (*t*(31) = –3.17, *p* = 0.01), of *environment*, with higher RTs in reversal environments (*F*(1,31) = 6.91, *p* = 0.01, $\eta_{p}^{2}=0.18$), and of *previous trial outcome*, with higher RTs if the previous trial did not match cue-target contingency (*F*(1,31) = 73.49, *p* < 0.001, $\eta_{p}^{2}=0.7$). In addition, the four-way interaction was significant (*F*(2,62) = 5.57, *p* = 0.006, $\eta_{p}^{2}=0.15$). Follow-up three-way ANOVAs stratified by previous trial outcome showed no prior-related interactions when previous trials matched the cue-target contingency (*p* = 0.6). Conversely, when previous trials did not match the cue-target contingency, the *block phase* x *environment* x *prior* interaction was significant (*F*(2,62) = 6.08, *p* = 0.004, $\eta_{p}^{2}=0.16$). This was driven by a *block phase* x *environment* interaction for true priors (*F*(2,62) = 9.71, *p* < 0.001, $\eta_{p}^{2}=0.24$), showing an increase in RTs from the first phase to the third phase when the previous trial did not match the cue-target contingency in the reversal environment (*t*(31) = –3.31, *p* = 0.004) and a decrease in the stable environment (*t*(31) = 2.41, *p* = 0.04) (Figure 2C). This pattern did not emerge for false priors (p = 0.15) (Figure 2D; see Figure S3 for RTs after previous trials that matched the cue-target contingency).

Taken together, the observed patterns in cueing effects on accuracy and RT support the conclusion that participants successfully learned and adapted to the cue-target contingencies and their changes. However, contrary to our initial hypothesis, primary analysis yielded no significant modulation of updating behavior by the prior volatility beliefs. Exploratory post-hoc analysis uncovered a prior effect limited to RTs of more frequent/expected trials following trials that did not match the cue-target contingency.

Consistent with the absence of prior effects in the primary model-free analyses, there was no evidence that the prior affected the learning rate *α* of the RW model. The a priori paired t-tests comparing learning rates within each environment showed no significant results, neither in the stable environment (*t*(31) = 0.01, *p* = 0.99) nor in the reversal environment (reversal: *t*(31) = 1.32, *p* = 0.2) (see Figure 2E). Likewise, in the 2 × 2 ANOVA on learning rate, neither the main effects nor the interaction reached significance (all *p* > 0.25) (see Figure 2E). Similarly, the exploratory analysis of the response model parameter *ζ*_2_ showed no effect of prior (stable environment: *t*(31) = –0.27, *p* = 0.79; reversal environment: *t*(31) = –0.15, *p* = 0.88).

### Predictive task

Similar to the reactive task, participants exhibited a high accuracy in the question trials (87 ± 3.7% SEM), suggesting that they attended to the information at the beginning of the blocks and could reliably identify false instructions. This was true regardless of condition, with a 2 × 2 ANOVA with the factors *environment* and *prior* not yielding any significant results.

**Figure 3:**
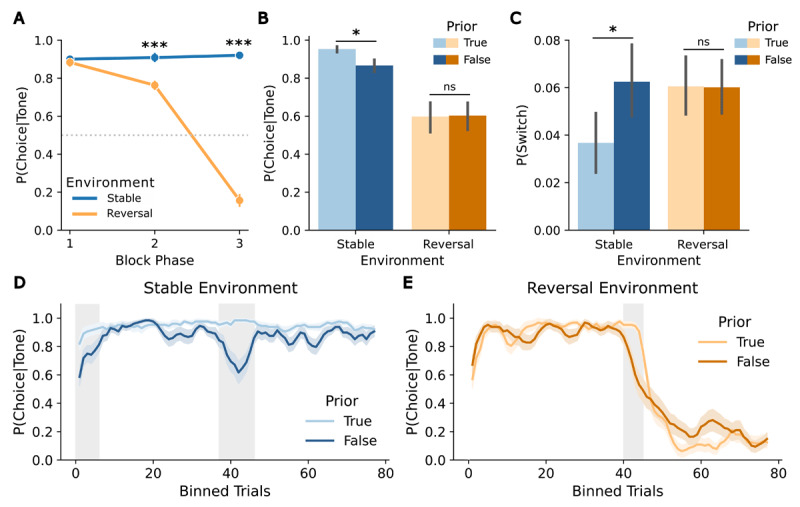
Adaptation to changes in cue-target contingencies and influence of prior volatility beliefs on model-free measures in the predictive task. **(A)** Participants’ choice accuracy across block phases in both environments (stable, reversal) demonstrates adaptation to the cue-target contingency. **(B)** Choice accuracy by environment (stable, reversal) and prior (true, false). **(C)** Probability of switching by environment (stable, reversal) and prior (true, false). **(D)** Choice accuracy in the stable environment, averaged using a sliding window (n = 4) separated by prior (true, false). **(E)** Choice accuracy in the reversal environment, averaged using a sliding window (n = 4) separated by prior (true, false). Asterisks indicate significance levels: p < 0.05 (*), p < 0.01 (**), p < 0.001 (***). Error bars reflect SEMs.

The analysis of the choice data using a 3 × 2 × 2 ANOVA with factors *block phase, environment*, and *prior* revealed a significant main effect of *block phase* (*F*(2,62) = 178.29, *p* < 0.001, $\eta_{p}^{2}=0.85$), with decreasing choice probability over the block phases (all post-hoc pairwise t-tests *p* < 0.006), a significant main effect of *environment*, showing higher choice probabilities in stable environments (*F*(1,31) = 203.41, *p* < 0.001, $\eta_{p}^{2}=0.89$), and a significant main effect of *prior*, indicating higher choice probability for true priors (*F*(1,31) = 11.29, *p* = 0.002, $\eta_{p}^{2}=0.27$). Moreover, a significant *block phase* x *environment* interaction was observed (*F*(2,62) = 184.33, *p* < 0.001, $\eta_{p}^{2}=0.86$). Post-hoc pairwise t-tests revealed the expected pattern, with differences between environments emerging in the second (*t*(31) = 5.19, *p* < 0.001) and third phase (*t*(31) = 17.45, *p* < 0.001) of a block. More specifically, choices remained consistent in the stable environment but changed in the reversal environment throughout the block (see Figure 3A), indicating choice adaptation to the change in the cue-target contingency. Furthermore, the *environment* x *prior* interaction was significant (*F*(1,31) = 6.4, *p* = 0.02, $\eta_{p}^{2}=0.17$). Post-hoc pairwise t-tests indicated that in a stable environment, participants’ target location predictions were more often correct when the prior was true than when it was false (*t*(31) = –4.32, *p* < 0.001) (Figure 3B). Additionally, the *block phase* x *prior* interaction was significant (*F*(2,62) = 7.03, *p* = 0.002, $\eta_{p}^{2}=0.19$). Post-hoc pairwise t-tests showed a decrease in choice probability when the prior was false in both environments during the second block phase (*t*(31) = –5.5, *p* < 0.001). The three-way interaction was not significant, showing that the effect of prior was similar across all block phases (p = 0.28).

In the analysis of the switching behavior (changes in choices after unexpected trials) the 3 × 2 × 2 ANOVA with factors *block phase, environment*, and *prior*, revealed a significant main effect of *prior*, showing a higher probability of switching for false priors (*F*(2,62) = 8.47, *p* = 0.007, $\eta_{p}^{2}=0.22$). Moreover, the *environment* x *prior* interaction was significant (*F*(2,62) = 7.34, *p* = 0.01, $\eta_{p}^{2}=0.19$). Post-hoc comparisons showed lower switching probability in the stable environment with a true prior than with a false prior (*t*(31) = 3.29, *p* = 0.02), but no difference between priors in reversal environments (*p* = 1) (Figure 3C). No other effects of the ANOVA were significant.

The cluster permutation analysis of the sliding-window choice data revealed significant differences in choice behavior between true and false priors in both environments. In the stable environment, a significant cluster was identified in the change expectation phase (trials 37–46: cluster-level *t* = 33.61, *p* = 0.001), indicating a drop in choice accuracy under a false (reversal) prior. Additionally, a cluster in the initial learning phase showed a trend towards significance (trials 0–5: cluster-level *t* = 14.21, *p* = 0.05), suggesting slower initial learning of the cue-target contingency with a false (volatility) prior (Figure 3D). In the reversal environment, a significant cluster was observed in the change expectation phase (trials 40–44: cluster-level *t* = 19.81, *p* = 0.03), indicating earlier adaptation to the reversal with a false (stable) prior (Figure 3E). Qualitatively, subsequent choices in the false prior condition tended to reach an asymptotic level later than with a true prior.

**Figure 4:**
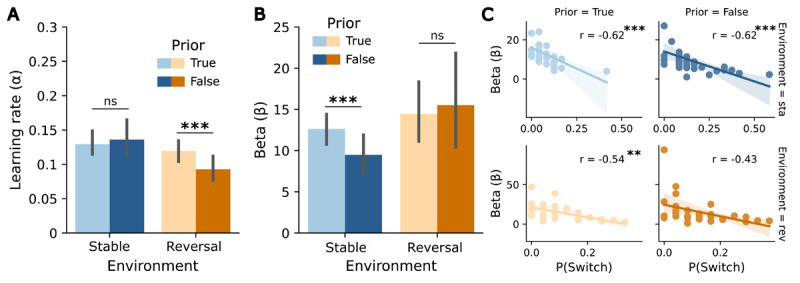
Influence of the prior volatility belief on the RW model parameters in the predictive task. **(A)** Learning rate *α* for both environments (stable, reversal) and priors (true, false). **(B)** *β*-values for both environments (stable, reversal) and priors (true, false). **(C)** Correlation plots between probability of switching and *β*-values by environment (stable, reversal) and prior (true, false). Asterisks indicate significance levels: p < 0.05 (*), p < 0.01 (**), p < 0.001 (***). Error bars reflect SEMs.

For the learning rate α, estimated from the RW model, within-environment t-tests showed a significant effect of the prior in the reversal environment (*t*(31) = 3.6, *p* = 0.001). Participants updated their beliefs faster when the prior was true than when it was false, in line with the assumption that stability beliefs lead to slower updating. In the stable environment, the difference between priors was not significant (*p* = 0.71) (see Figure 4A). The 2 × 2 ANOVA revealed a significant main effect of *environment* (*F*(1,31) = 6.78, *p* = 0.014, $\eta_{p}^{2}=0.18$), with higher learning rates for the stable environment, but no significant *prior x environment* interaction (*p* = 0.13) (see Figure 4A). The exploratory analysis of the response parameter *β* yielded a significant effect of the prior in the stable environment (*t*(31) = 3.302, *p* = 0.002), with higher *β*-values for true priors, indicating that responses in the stable environment were less noisy under stability assumptions (see Figure 4B). There was no effect in the reversal environment (*p* = 0.74). *β*-values were significantly negatively correlated with the probability of switching in three of the four conditions (true-stable: *r* = –0.62, *p* < 0.001; false-stable: *r* = –0.62, *p* < 0.001; true-reversal: *r* = –0.54, *p* = 0.008). In the reversal environment with a false prior, the negative correlation did not remain significant after Bonferroni correction (*r* = –0.43, *p* = 0.06). These results suggest that participants with higher *β*-values tended to show lower probabilities of switching, especially under stable and true prior conditions (see Figure 4C).

In sum, the observed patterns in choice data in the predictive task showed (as for the reactive task) that participants successfully learned the cue-target contingency and adapted to these associations and their changes. In contrast to the primary analysis of the reactive task, we observed effects of the prior volatility belief: In reversal environments, false priors decreased the learning rate, as predicted. In stable environments, false priors affected decision noise, leading to reduced choice accuracy and a higher probability of switching.

### Task comparison

**Figure 5:**
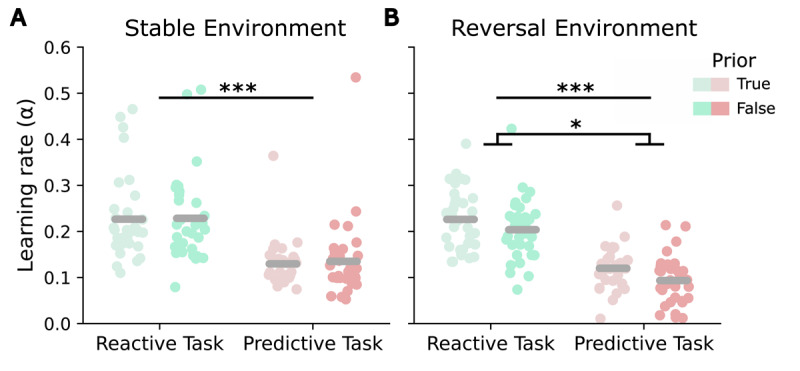
Learning rates in the reactive as compared to the predictive task. **(A)** Learning rates *α* for true and false priors per task in a stable environment. **(B)** Learning rates *α* for true and false priors per task in a reversal environment. Asterisks indicate significance levels: p < 0.05 (*), p < 0.01 (**), p < 0.001 (***).

The comparison of the RW learning rates from both task versions using a 2 × 2 ANOVA with the factors *task version* and *prior* (separately for stable and reversal environment) showed a higher learning rate for the reactive task compared to the predictive task for both environments (main effect of *task version*: stable environment: *F*(1,31) = 38.87, *p* < 0.001, $\eta_{p}^{2}=0.57$, Figure 5A; reversal environment: *F*(1,31) = 135.3, *p* < 0.001, $\eta_{p}^{2}=0.81$, Figure 5B). Additionally, in the reversal environment, a significant main effect of *prior* was detected (*F*(1,31) = 5.95, *p* = 0.04, $\eta_{p}^{2}=0.16$), indicating lower learning rates when the prior was false. There were no significant interaction effects in either environment (stable environment: *p* = 0.81; reversal environment: *p* = 0.85). No significant correlations between the learning rates of both tasks were identified under any condition.

## Discussion

While previous research has shown that learning rates depend on experienced volatility (Behrens et al., 2007), it remains unclear how *prior* beliefs about volatility impact updating behavior, particularly when empirical observations violate these beliefs. To explore this, we implemented a cueing/reversal learning task with true or false prior volatility information in both reactive and predictive task versions. Our results suggest that the task requirements modulate the effects of prior volatility information. When participants were required to react to a target stimulus based on its location (reactive task), prior information did not significantly affect updating of cue-target contingencies in the main analyses. In contrast, when participants were required to predict the target’s location before it appeared (predictive task), prior beliefs impacted both model-free and model-based signatures of updating and response adaptation. More specifically, in the predictive task version, false beliefs of stability decreased the learning rate in a reversal environment. While there was no learning rate modulation in the stable environment, the false belief of volatility increased decision noise and switching behavior. In general, learning rates were higher in the reactive than in the predictive task version.

### Prior volatility beliefs shape updating for active predictions

Adaptive behavior depends on continuously adjusting the balance between maintaining stable beliefs and remaining flexible to change (Leimar et al., 2024; Nassar & Troiani, 2021; Pulcu & Browning, 2019). In the context of our probabilistic reversal learning task, we hypothesized that prior beliefs about environmental volatility would bias this flexibility-stability trade-off by modulating learning rates (with slower updating under stability assumptions and faster updating under volatility assumptions) based on previous work (e.g., Iglesias et al., 2013; Liu et al., 2023). Consistent with this, in reversal environments, a false prior led to lower learning rates in the predictive task, indicating slower updating when participants incorrectly believed the environment was stable. This finding aligns with previous work showing that learning rates are sensitive to perceived environmental volatility (Behrens et al., 2007; Iglesias et al., 2013) and extends these findings by demonstrating that *prior* higher-order volatility beliefs can affect adaptive behavior, even when sensory evidence contradicts these priors.

Interestingly, although the true prior was associated with a higher learning rate in the reversal environment, this was not directly reflected in the model-free cluster-permutation analysis of the choices. This analysis showed that participants tended to start adapting to the reversal earlier when they expected stability than when they expected volatility. Still, in line with a higher learning rate, subsequent choices descriptively appeared to reach an asymptotic level earlier with a true prior than with a false prior (although no significant clusters were detected in this later phase). Hence, one could argue that this difference in adaptation may have been better captured in the model-based analysis. The dissociation between learning rate and earlier behavioral adaptation may suggest that flexibility can come at a cost: while higher learning rates can facilitate rapid adaptation, they also increase susceptibility to noise (Nassar & Troiani, 2021; Soltani & Izquierdo, 2019). Supporting this interpretation, Liu et al. (2023) showed that theta-band transcranial alternating current stimulation over the dorsolateral prefrontal cortex led to increased learning rates but impaired performance in a probabilistic learning task, particularly in a more stable environment. In our study, expecting stability may have allowed participants to focus more on the overall change in cue-target contingencies rather than being distracted by trial-by-trial fluctuations driven by the expectation of change.

### Environmental context determines how false priors manifest for active predictions

We expected prior beliefs to modulate learning rates in both stable and reversal environments. However, in contrast to the reversal environment, we did not observe a significant difference in learning rates for true and false priors in the stable environment of the predictive task. Instead, further analysis in this environment revealed a modulation of the decision noise parameter *β* of the response model. Participants were more exploratory in their choices when the prior was false (i.e., when they expected volatility), which was accompanied by increased probability of switching and lower choice accuracy. This observation suggests that in stable environments, the effect of false priors may not necessarily be captured by changes in the learning rate, but instead manifests in decision noise.

In a similar vein, a recent study reported that expecting volatility increased the decision noise, further supporting this idea (Jedlovszky et al., 2024). Notably, Jedlovszky et al. (2024) did not observe any modulation of learning rates by volatility or stability beliefs. The authors speculated that this could be due to a lack of uncertainty about volatility, since participants were not explicitly told that volatility levels could change. In our design, volatility expectations may have been more salient due to the more specific instructions about the presence/absence of a change and the question at the end of each block, which may have increased their impact on belief updating. This may explain why we observed a modulation of the learning rate in the reversal environment.

Still, while increased salience may explain why we observed an effect in the reversal environment, unlike Jedlovszky et al. (2024), it does not account for the absence of learning rate effects in the stable environment. This missing modulation may be attributed to the way the environment was structured. Unlike previous studies, where the stable environment still included reversals (Jedlovszky et al., 2024; Simoens et al., 2024), our stable environment was entirely free of reversals, whereas our reversal environment included only one contingency change. This design allowed us to isolate the impact of volatility expectations in a truly stable setting. Moreover, it ensured that participants had sufficient trials to infer and adapt to the contingencies, while keeping the experiment duration within tolerable limits. The lack of reversals in stable blocks also meant that participants were unlikely to experience substantial prediction errors. As learning in the Rescorla-Wagner framework is regulated by the magnitude of prediction errors (weighted by the learning rate), the lower magnitude of such errors in our design likely decreased the need for belief updating. Therefore, even when participants expected volatility, there was no measurable adjustment of learning rates. Given these differences in task structure and results between this study and previous studies, future research should further explore how varying levels of volatility and the uncertainty associated with them influence the effect of prior beliefs.

### Task demands shape the speed of belief updating and the modulation by prior information

Contrary to our prediction that prior beliefs would modulate learning rates in both task versions, we did not observe a strong effect of prior volatility beliefs in our primary analysis in the reactive task. However, two observations suggest a subtle influence of the volatility prior. First, a post-hoc analysis revealed a modulation of RTs depending on prior and environment in expected trials following a trial that did not match the cue-target contingency. Second, we did observe a modulation of the learning rate in reversal blocks in the analysis combining both tasks (a main effect of prior, without a significant interaction with task). Taken together, these observations indicate that the influence of prior was not absent, but less pronounced and more variable in the reactive task.

A plausible explanation is that this pattern reflects differences in task demands. Behavior in the reactive task is more stimulus-driven, i.e., shaped primarily by observed sensory input rather than higher-order beliefs. In contrast, the predictive task requires participants to actively use their beliefs to select a response before observing the outcome, making it potentially more dependent on goal-directed processes that may be more prone to beliefs (especially higher-order beliefs such as volatility).

At a more global level, these task-demand differences were also reflected in overall belief updating speed. When comparing the two tasks of our study, learning rates were higher in the reactive task. This result is in line with our hypothesis. Previous work has shown that interacting with the environment can create a stronger sense of stability and slower updating compared to passive observation (Weiss et al., 2021; Yon et al., 2023). Extending this line of work, we report that explicit predictions, despite the absence of instrumental control over outcomes, can similarly promote more stable belief updating. Consistent with the task-demand interpretation above, participants may have relied on more immediate sensory evidence in the reactive task, leading to more rapid belief updates. The absence of explicit predictions may also have reduced participants’ perceived control over the environment. Lower perceived control can increase sensitivity to environmental changes, as individuals may feel they have less influence over outcomes and therefore perceive the environment as less stable (Bhanji & Delgado, 2014). Taken together, these findings suggest that the behavioral impact of prior beliefs about volatility depends on the task context in which those beliefs are expressed. By combining predictive and reactive responses with true and false prior information, the present study sheds light on the factors that influence how prior volatility beliefs affect adaptive behavior.

### Limitations

A limitation of our present study is that our reversal blocks reflected a relatively mild form of volatility (containing only a single reversal) compared with previously used paradigms that included multiple reversals. This design was chosen to present enough trials so that participants could learn the probabilistic cue-target association and experience the reversal. At the same time, multiple short blocks were needed to manipulate prior beliefs through instructions, limiting the number of reversals. However, this may have reduced the differences in learning rates between the two environments, since differences between the two block types emerged only in the second half of the block. As a consequence, the design may have had limited sensitivity to detect environment-specific differences in the learning rate modulation by prior. Consistent with this, the prior-by-environment interaction was not significant in an ANOVA including environment as a factor. Accordingly, the present findings emerged only when the effects of higher-order volatility beliefs were contrasted within environments. Future work should test whether the present pattern holds under more pronounced volatility manipulations.

### Implications and conclusion

Taken together, our findings demonstrate a significant impact of prior volatility beliefs on adaptive behavior. Moreover, they highlight the importance of task demands when investigating the influence of priors on updating. These results are particularly relevant for understanding maladaptive volatility beliefs in neuropsychiatric conditions: a false belief that persists despite contradictory evidence can lead to inappropriate adjustments in learning rates, ultimately resulting in maladaptive behavioral patterns, such as an inability to adapt to change in anxious individuals and those at high risk of psychosis (Browning et al., 2015; Cole et al., 2020) or an overestimation of volatility in individuals with autism (Lawson et al., 2017). By elucidating how and under what conditions higher-order beliefs influence learning processes, our study offers valuable insights into the mechanisms underlying adaptive behavior.

## Additional File

The additional file for this article can be found as follows:

10.5334/joc.504.s1 Supplementary Materials. The supplementary materials provide expanded results and further details on the computational modelling, complementing the main text.

## Data Availability

The raw data and supplementary materials for this study will be available at https://doi.org/10.26165/JUELICH-DATA/8KTGAU upon publication.

## References

[1] Bağci, B., Düsmez, S., Zorlu, N., Bahtiyar, G., Isikli, S., Bayrakci, A., Heinz, A., Schad, D. J., & Sebold, M. (2022). Computational analysis of probabilistic reversal learning deficits in male subjects with alcohol use disorder. Frontiers in Psychiatry, 13. 10.3389/fpsyt.2022.960238 PMC9626515 36339830

[2] Behrens, T. E. J., Woolrich, M. W., Walton, M. E., & Rushworth, M. F. S. (2007). Learning the value of information in an uncertain world. Nature Neuroscience, 10(9), Article 9. 10.1038/nn1954 17676057

[3] Bhanji, J. P., & Delgado, M. R. (2014). Perceived Control Influences Neural Responses to Setbacks and Promotes Persistence. Neuron, 83(6), 1369–1375. 10.1016/j.neuron.2014.08.012 25199702 PMC4169331

[4] Browning, M., Behrens, T. E., Jocham, G., O’Reilly, J. X., & Bishop, S. J. (2015). Anxious individuals have difficulty learning the causal statistics of aversive environments. Nature Neuroscience, 18(4), 590–596. 10.1038/nn.3961 25730669 PMC4644067

[5] Cole, D. M., Diaconescu, A. O., Pfeiffer, U. J., Brodersen, K. H., Mathys, C. D., Julkowski, D., Ruhrmann, S., Schilbach, L., Tittgemeyer, M., Vogeley, K., & Stephan, K. E. (2020). Atypical processing of uncertainty in individuals at risk for psychosis. NeuroImage: Clinical, 26, 102239. 10.1016/j.nicl.2020.102239 32182575 PMC7076146

[6] Frässle, S., Aponte, E. A., Bollmann, S., Brodersen, K. H., Do, C. T., Harrison, O. K., Harrison, S. J., Heinzle, J., Iglesias, S., Kasper, L., Lomakina, E. I., Mathys, C., Müller-Schrader, M., Pereira, I., Petzschner, F. H., Raman, S., Schöbi, D., Toussaint, B., Weber, L. A., … Stephan, K. E. (2021). TAPAS: An Open-Source Software Package for Translational Neuromodeling and Computational Psychiatry. Frontiers in Psychiatry, 12. 10.3389/fpsyt.2021.680811 PMC8206497 34149484

[7] Friston, K. (2010). The free-energy principle: A unified brain theory? Nature Reviews Neuroscience, 11(2), 127–138. 10.1038/nrn2787 20068583

[8] Gagne, C., Zika, O., Dayan, P., & Bishop, S. J. (2020). Impaired adaptation of learning to contingency volatility in internalizing psychopathology. eLife, 9, e61387. 10.7554/eLife.61387 33350387 PMC7755392

[9] Gramfort, A., Luessi, M., Larson, E., Engemann, D. A., Strohmeier, D., Brodbeck, C., Goj, R., Jas, M., Brooks, T., Parkkonen, L., & Hämäläinen, M. (2013). MEG and EEG data analysis with MNE-Python. Frontiers in Neuroscience, 7–2013. 10.3389/fnins.2013.00267 PMC3872725 24431986

[10] Hauke, D. J., Wobmann, M., Andreou, C., Mackintosh, A. J., de Bock, R., Karvelis, P., Adams, R. A., Sterzer, P., Borgwardt, S., Roth, V., & Diaconescu, A. O. (2024). Altered Perception of Environmental Volatility During Social Learning in Emerging Psychosis. Computational Psychiatry, 8(1), 1–22. 10.5334/cpsy.95 38774429 PMC11104374

[11] Hein, T. P., de Fockert, J., & Ruiz, M. H. (2021). State anxiety biases estimates of uncertainty and impairs reward learning in volatile environments. NeuroImage, 224, 117424. 10.1016/j.neuroimage.2020.117424 33035670

[12] Iglesias, S., Mathys, C., Brodersen, K. H., Kasper, L., Piccirelli, M., DenOuden, H. E. M., & Stephan, K. E. (2013). Hierarchical Prediction Errors in Midbrain and Basal Forebrain during Sensory Learning. Neuron, 80(2), Article 2. 10.1016/j.neuron.2013.09.009 24139048

[13] Izquierdo, A., Brigman, J. L., Radke, A. K., Rudebeck, P. H., & Holmes, A. (2017). The neural basis of reversal learning: An updated perspective. Cognitive Flexibility: Development, Disease, and Treatment, 345, 12–26. 10.1016/j.neuroscience.2016.03.021 PMC5018909 26979052

[14] Jedlovszky, K., Corlett, P. R., & Yon, D. (2024). Subjective Volatility, Learning and Paranoia (Version 1). PsyArXiv. 10.31234/osf.io/sre9y

[15] Lawson, R. P., Mathys, C., & Rees, G. (2017). Adults with autism overestimate the volatility of the sensory environment. Nature Neuroscience, 20, 1293–1299. 10.1038/nn.4615 28758996 PMC5578436

[16] Leimar, O., Quiñones, A. E., & Bshary, R. (2024). Flexible learning in complex worlds. Behavioral Ecology, 35(1), arad109. 10.1093/beheco/arad109 38162692 PMC10756056

[17] Liu, M., Dong, W., Wu, Y., Verbeke, P., Verguts, T., & Chen, Q. (2023). Modulating hierarchical learning by high-definition transcranial alternating current stimulation at theta frequency. Cerebral Cortex, 33(8), 4421–4431. 10.1093/cercor/bhac352 36089836

[18] Mathys, C., Daunizeau, J., Friston, K. J., & Stephan, K. E. (2011). A bayesian foundation for individual learning under uncertainty. Frontiers in Human Neuroscience, 5(39). 10.3389/fnhum.2011.00039 PMC3096853 21629826

[19] Mengotti, P., Dombert, P. L., Fink, G. R., & Vossel, S. (2017). Disruption of the Right Temporoparietal Junction Impairs Probabilistic Belief Updating. The Journal of Neuroscience : The Official Journal of the Society for Neuroscience, 37(22), Article 22. 10.1523/JNEUROSCI.3683-16.2017 PMC6596530 28473647

[20] Mengotti, P., Käsbauer, A.-S., Fink, G. R., & Vossel, S. (2022). Combined TMS-fMRI Reveals Behavior-Dependent Network Effects of Right Temporoparietal Junction Neurostimulation in an Attentional Belief Updating Task. Cerebral Cortex, 32(21), 4698–4714. 10.1093/cercor/bhab511 35088068

[21] Microsoft Corporation. (n.d.). Visual Studio Code. Retrieved https://code.visualstudio.com/

[22] Nassar, M. R., & Troiani, V. (2021). The stability flexibility tradeoff and the dark side of detail. Cognitive, Affective, & Behavioral Neuroscience, 21(3), 607–623. 10.3758/s13415-020-00848-8 PMC8141540 33236296

[23] Palminteri, S., Wyart, V., & Koechlin, E. (2017). The Importance of Falsification in Computational Cognitive Modeling. Trends in Cognitive Sciences, 21(6), 425–433. 10.1016/j.tics.2017.03.011 28476348

[24] Piray, P., & Daw, N. D. (2020). A simple model for learning in volatile environments. PLOS Computational Biology, 16(7), e1007963. 10.1371/journal.pcbi.1007963 32609755 PMC7329063

[25] Posner, M. I. (1980). Orienting of Attention. Quarterly Journal of Experimental Psychology, 32(1), 3–25. 10.1080/00335558008248231 7367577

[26] Pulcu, E., & Browning, M. (2019). The Misestimation of Uncertainty in Affective Disorders. Trends in Cognitive Sciences, 23(10), 865–875. 10.1016/j.tics.2019.07.007 31431340

[27] Reed, E. J., Uddenberg, S., Suthaharan, P., Mathys, C. D., Taylor, J. R., Groman, S. M., & Corlett, P. R. (2020). Paranoia as a deficit in non-social belief updating. eLife, 9, e56345. 10.7554/eLife.56345 32452769 PMC7326495

[28] Rescorla, R. A., & Wagner, A. R. (1972). A theory of Pavlovian conditioning: Variations in the effectiveness of reinforcement. In Classical Conditioning II: Current Research and Theory (pp. 64–99). Appleton-Century-Crofts.

[29] Sandhu, T. R., Xiao, B., & Lawson, R. P. (2023). Transdiagnostic computations of uncertainty: Towards a new lens on intolerance of uncertainty. Neuroscience & Biobehavioral Reviews, 148, 105123. 10.1016/j.neubiorev.2023.105123 36914079

[30] Schiffer, A. M., Siletti, K., Waszak, F., & Yeung, N. (2017). Adaptive behaviour and feedback processing integrate experience and instruction in reinforcement learning. NeuroImage, 146, 626–641. 10.1016/j.neuroimage.2016.08.057 27577720 PMC5312784

[31] Simoens, J., Verguts, T., & Braem, S. (2024). Learning environment-specific learning rates. PLOS Computational Biology, 20(3), e1011978. 10.1371/journal.pcbi.1011978 38517916 PMC10990245

[32] Soltani, A., & Izquierdo, A. (2019). Adaptive learning under expected and unexpected uncertainty. Nature Reviews Neuroscience, 20(10), Article 10. 10.1038/s41583-019-0180-y PMC6752962 31147631

[33] Stephan, K. E., Penny, W. D., Daunizeau, J., Moran, R. J., & Friston, K. J. (2009). Bayesian model selection for group studies. NeuroImage, 46(4), 1004–1017. 10.1016/j.neuroimage.2009.03.025 19306932 PMC2703732

[34] Topel, S., Ma, I., Sleutels, J., van Steenbergen, H., de Bruijn, E. R. A., & van Duijvenvoorde, A. C. K. (2023). Expecting the unexpected: A review of learning under uncertainty across development. Cognitive, Affective, & Behavioral Neuroscience, 23(3), 718–738. 10.3758/s13415-023-01098-0 PMC10390612 37237092

[35] Vossel, S., Mathys, C., Daunizeau, J., Bauer, M., Driver, J., Friston, K. J., & Stephan, K. E. (2014). Spatial Attention, Precision, and Bayesian Inference: A Study of Saccadic Response Speed. Cerebral Cortex, 24(6), 1436–1450. 10.1093/cercor/bhs418 23322402 PMC4014178

[36] Weiss, A., Chambon, V., Lee, J. K., Drugowitsch, J., & Wyart, V. (2021). Interacting with volatile environments stabilizes hidden-state inference and its brain signatures. Nature Communications, 12(1), 2228. 10.1038/s41467-021-22396-6 PMC8044147 33850124

[37] Yon, D., Thomas, E. R., Gilbert, S. J., de Lange, F. P., Kok, P., & Press, C. (2023). Stubborn Predictions in Primary Visual Cortex. Journal of Cognitive Neuroscience, 35(7), 1133–1143. 10.1162/jocn_a_01997 37083997

