# Supplementary material for: The Impact of Prior Beliefs about Volatility on Adaptive Behavior
Source: J Cogn. 2026 May 27;9(1):32. doi: 10.5334/joc.504 (PMC13220732; doi:10.5334/joc.504)
Supplement: Supplementary Materials. — The supplementary materials provide expanded results and further details on the computational modelling, complementing the main text. [file joc-9-1-504-s1.pdf]

Supplementary Materials to

Bleser et al. The Impact of Prior Beliefs about Volatility on Adaptive Behavior

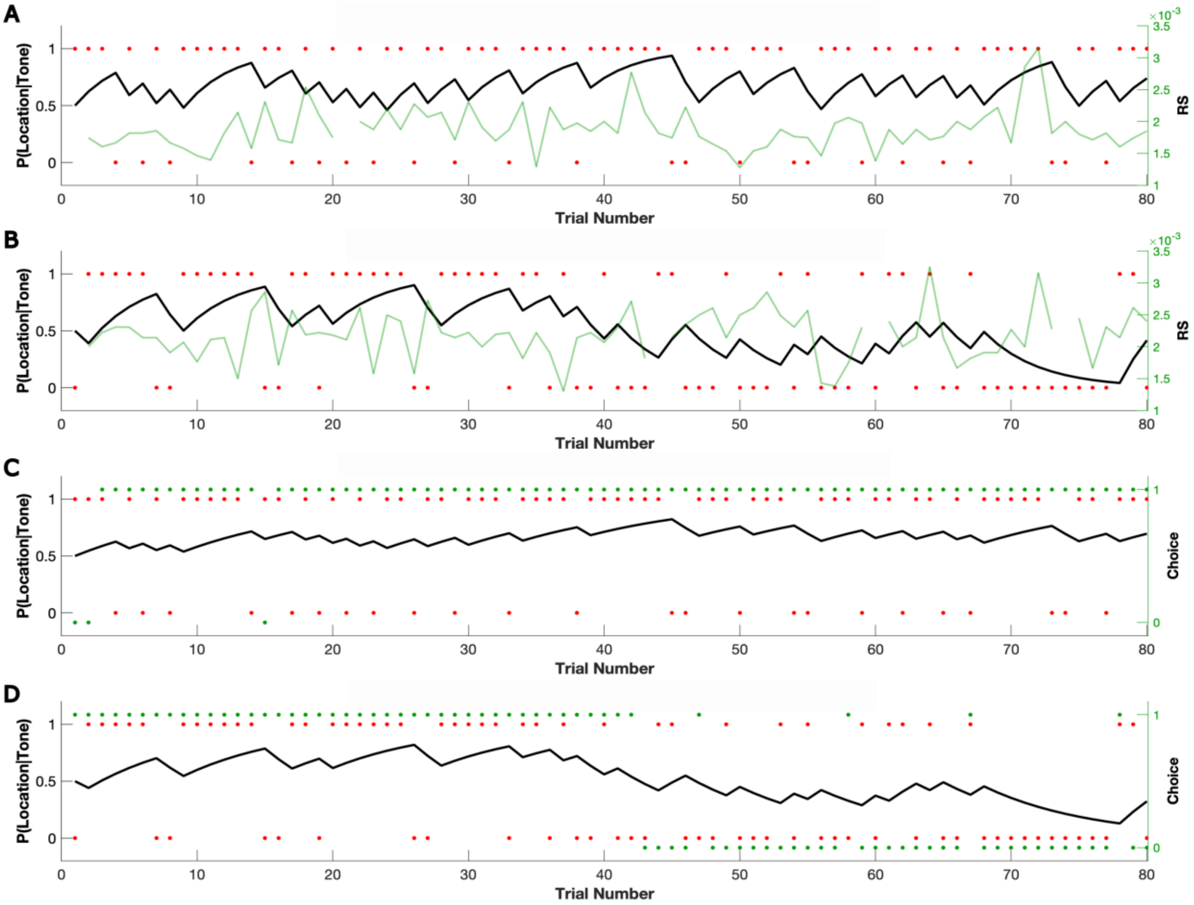

Figure S1. Example trajectories of Rescorla Wagner model fit from single participants in the sample. Panels show representative participant estimates of P(location=1|tone=1) across trials for different task versions and conditions. **(A)** Reactive task, stable environment. **(B)** Reactive task, reversal environment. **(C)** Predictive task, stable environment. **(D)** Predictive task, reversal environment. Red dots represent whether trials within this block are expected (outcome matches cue-target contingency of the first half, outcome = 1) or unexpected (outcome does not match cue-target contingency of the first half, outcome = 0). In panels A and B, the participant's response speed (1/RT (ms)) is shown as a green line. In panels C and D, the participant's choices are shown as green dots.

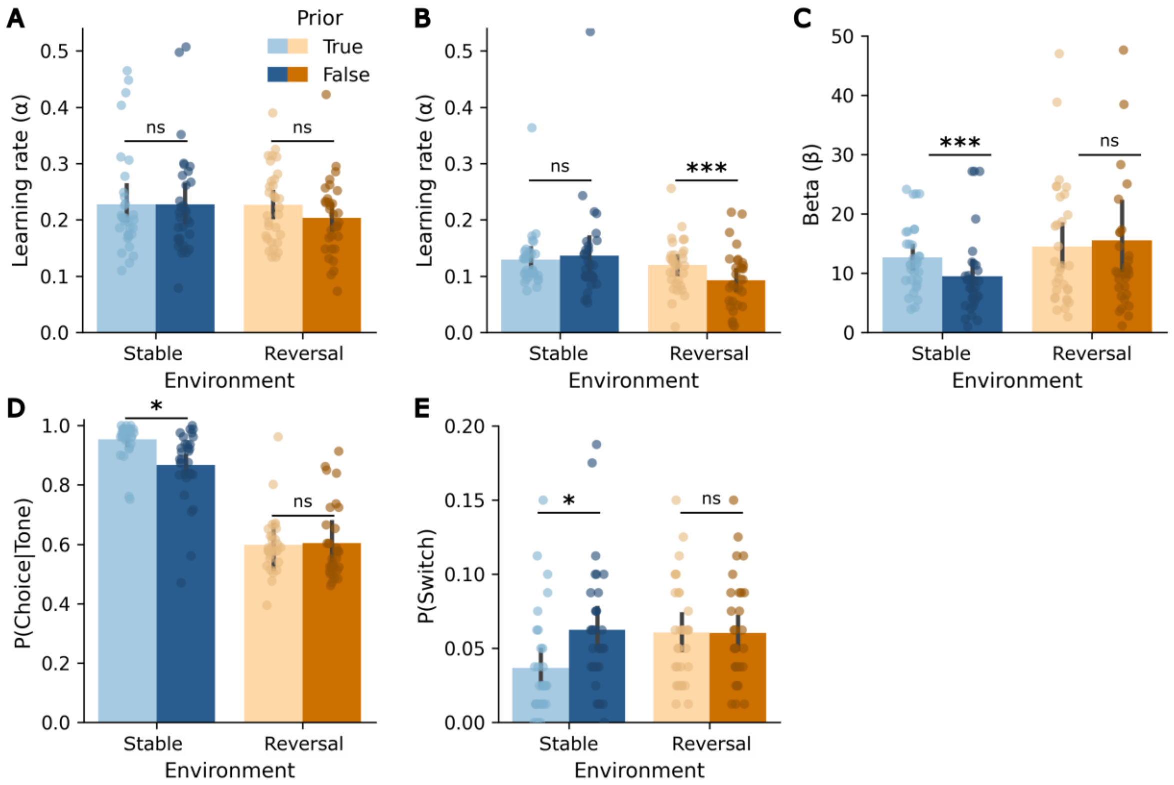

Figure S2. Expanded versions of the main figures with overlaid individual subject data. **(A)** Learning rate α for both environments (stable, reversal) and priors (true, false) in the reactive task. **(B)** Learning rate α for both environments (stable, reversal) and priors (true, false) in the predictive task. **(C)** Decision noise values β for both environments (stable, reversal) and priors (true, false) in the predictive task. **(D)** Choice accuracy by environment (stable, reversal) and prior (true, false) in the predictive task. **(E)** Probability of switching by environment (stable, reversal) and prior (true, false) in the predictive task. Asterisks indicate significance levels: p<.05 (*), p<.01 (**), p<.001 (***). Error bars reflect SEMs.

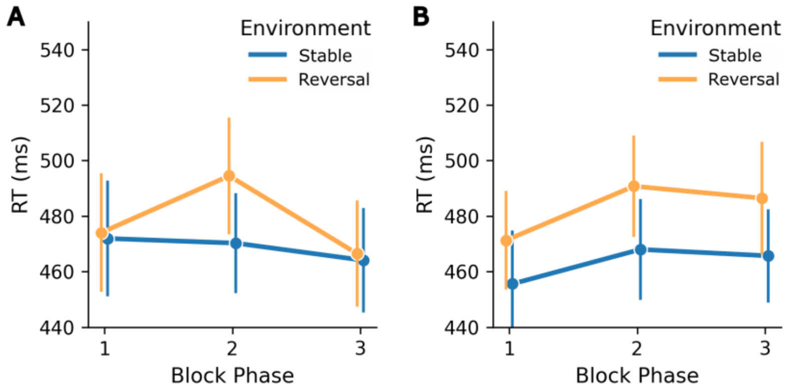

Figure S3. Post-hoc analysis in the reactive task. Expected trial RTs following a trial that matched the cue-target contingency shown separately for **(A)** true priors and **(B)** false priors. Error bars reflect SEMs.

Part A: Hierarchical Gaussian Filter description

The Hierarchical Gaussian Filter (HGF) is a Bayesian model of learning that formally describes how probabilistic beliefs are updated based on observations. It comprises a hierarchy of coupled Gaussian random walks. Each level in this hierarchy represents a hidden state that evolves over time. The lowest level tracks observable outcomes (i.e., if the target appeared on the expected or unexpected side in the present study), the middle level represents beliefs about those outcomes (i.e., cue-target contingencies), and the highest level estimates the volatility of the contingency changes. At each level, beliefs are updated based on prediction errors from the level below, with these errors being weighted by the precision of the current estimates. This precision-weighted updating allows the model to adapt flexibly according to environmental volatility. Key parameters include subject- and condition-specific parameters that control the variance of state changes at each level ($\square_2$ for the second and $\square_3$ for the third level). Modelling was performed using the HGF toolbox (7.0) as described by Mathys et al. (2011, 2014).

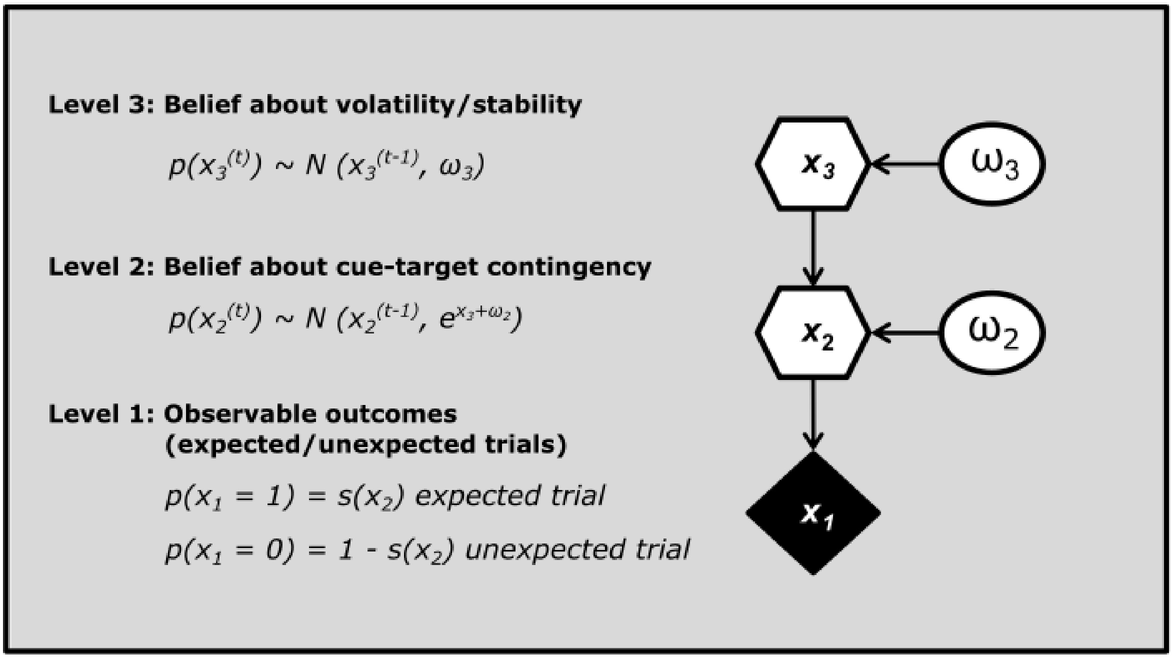

Figure S4. Schematic representation of the perceptual model of the Hierarchical Gaussian Filter (HGF) model. The model includes a hierarchy of states x_1, x_2 and x_3. The omega parameters (□$_2$ and □$_3$) reflect the rate at which subjects update their beliefs about the respective state x and are estimated from subjects' responses (response speed in the reactive task, choices in the predictive task). Circles denote constant values, diamonds represent time-varying values, and hexagons represent time-varying values that depend on their previous state. This perceptual model is combined with task-specific response models (as described in the Methods section) to generate observed responses.

Part B: Bayesian Model Selection (BMS) results

We used random-effects BMS (Stephan et al., 2009) to compare three alternative perceptual models. Bayesian model selection (BMS) accounts for model complexity (i.e., the number of adjustable parameters) and aims to identify models that generalize well to new data by avoiding overfitting.

The model space included the following models:

- RW: a standard Rescorla-Wagner model, with only one parameter (□) representing a fixed learning rate
- HGF3: a three-level HGF as described in section A

- □ HGF2: a reduced version of the three-level HGF where the third level of the hierarchy was removed by fixing both the log-volatility of the third level and the value of kappa to zero.

Among the three models tested, BMS favored the RW model. Table S1 reports the posterior probabilities (PP) and unprotected exceedance probabilities (XP) for each model. The exceedance probability reflects how likely it is that a particular model has a higher PP than any of the alternatives (see Stephan et al., 2009 for explanation).

Table S1. Posterior probabilities (PP) and exceedance probabilities (XP) for all models for both tasks.

	Reactive Task		Predictive Task	
BMS results	PP	XP	PP	XP
RW	0.99	1	0.87	0.99
HGF3	0.005	0	0.06	0.001
HGF2	0.005	0	0.07	0.004

Tables S2 and S3 summarize the means and standard deviations (SD) of the estimated parameters from the RW model, calculated across participants for the reactive task and the predictive task, respectively.

Table S2. Posterior estimates of the parameters estimated from the RW model for the reactive task.

	ST		SF		RT		RF	
Reactive task	Mean	SD	Mean	SD	Mean	SD	Mean	SD
□	0.23	0.09	0.23	0.09	0.23	0.06	0.2	0.07
ζ_1	0.002	3.9x10^{-4}	0.002	3.6x10^{-4}	0.002	3.9x10^{-4}	0.002	3.7x10^{-4}
ζ_2	5.8x10^{-4}	2.2x10^{-4}	5.9x10^{-4}	2.2x10^{-4}	5.5x10^{-4}	1.4x10^{-4}	5.5x10^{-4}	1.8x10^{-4}

ST = stable/true, SF = stable/false, RT = reversal/true, RF = reversal/false.

Table S3. Posterior estimates of the parameters estimated from the RW model for the predictive task.

	ST		SF		RT		RF	
Predictive task	Mean	SD	Mean	SD	Mean	SD	Mean	SD
□	0.13	0.05	0.14	0.09	0.12	0.05	0.09	0.05
β	12.62	5.58	9.47	6.84	14.44	10.45	15.53	17.51

ST = stable/true, SF = stable/false, RT = reversal/true, RF = reversal/false.

Table S4. Priors (mean and variance) used to estimate the RW model in both tasks.

	Mean	Variance
□	logit(0.2)	0.5
ζ_1 (reactive task)	log(0.002)	1
ζ_2 (reactive task)	log(0.0005)	0.1
β (predictive task)	log(3.9749)	1

Part C: Model validation

To assess whether the RW model could capture behavioral patterns observed at the group level, we simulated responses using the parameters estimated from the empirical data and applied the same model-free behavioral analyses to the simulated responses as to the observed data (Palminteri et al., 2017). Only the key effects identified in the empirical data are reported here for the simulated data. For analyses using an ANOVA, a 3 x 2 x 2 design was employed with factors *block phase*, *environment*, and *prior.* In the reactive task, this ANOVA revealed a significant interaction of *block phase* and *environment* for RT cueing effects (F(1.45,44.85)=7.68, p=0.003, η_p^2=0.2) (Figure S5 A) and for accuracy cueing effects (F(2,62)=5.08, p=0.009, η_p^2=0.14) (Figure S5 B), mirroring the observed behavioral patterns.

In the predictive task, the same ANOVA on simulated choice behavior revealed a significant *block phase* x *environment* interaction (F(1.58,48.9)=426.86, p<0.001, η_p^2=0.93) (Figure S5 C), as well as an *environment* x *prior* interaction (F(1,31)=4.65, p=0.04, η_p^2=0.13) (Figure S5 D). Applying the ANOVA to simulated switching behavior indicated the significant *environment* x *prior* interaction (F(1,31)=6.12, p=0.02, η_p^2=0.17) (Figure S5 E). The cluster permutation analysis on the sliding window choice data showed clusters that are

comparable to those observed in the empirical data. In the stable environment significant clusters were identified in trials 0-5 (cluster-level *t*=20.28, *p*=0.02) and trials 35-44 (cluster-level *t*=47.23, *p*<0.001). An additional significant cluster was observed for trials 49-63 (cluster-level *t*=57.3, *p*<0.001) (Figures S5 F). In the reversal environment, a significant cluster was observed in trials 38-44 (cluster-level *t*=26.34, *p*=0.004) An additional cluster was observed for trials 18-16 (cluster-level *t*=-31.81, *p*=0.002) (Figure S5 G).

Together, these analyses indicate that the predicted responses generated from the RW model using parameters estimated from the empirical data capture key behavioral patterns observed across both reactive and predictive tasks.

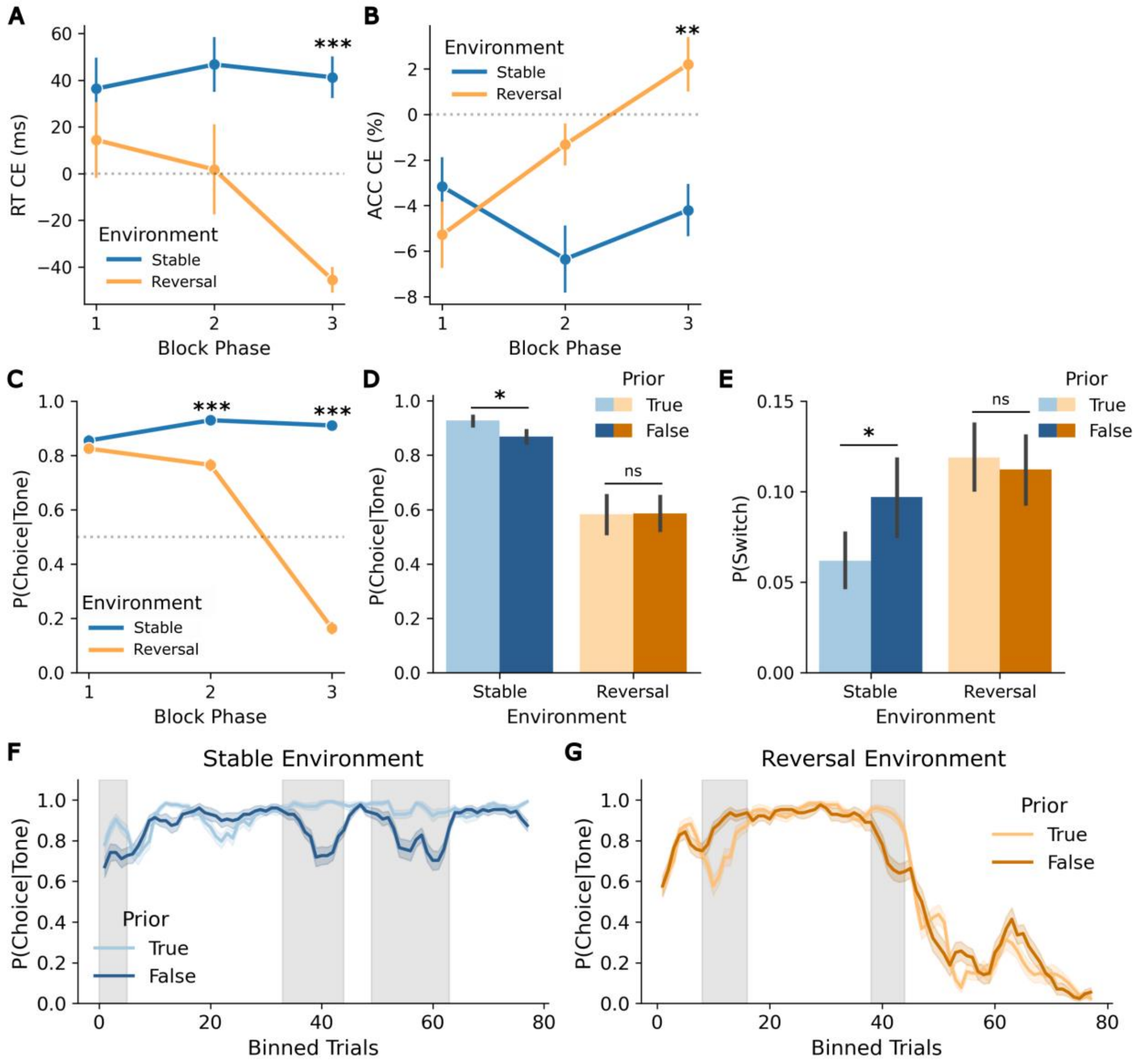

Figure S5. Results of model-free analyses on data simulated from estimated parameters in the reactive task (A & B) and the predictive task (C-G). **(A)** RT CEs (RT unexpected – RT expected, based on the contingency in the first half). **(B)** Accuracy CEs (ACC unexpected – ACC expected, based on the contingency in the first half). **(C)** Choice accuracy over block phases for both environments (stable, reversal). **(D)** Choice accuracy by environment (stable, reversal) and prior (true, false). **(E)** Probability of switching by environment (stable, reversal) and prior (true, false). **(F)** Choice accuracy in the stable environment, averaged using a sliding window (n=4) separated by prior (true, false). **(G)** Choice accuracy in the reversal environment, averaged using a sliding window (n=4) separated by prior (true, false). Asterisks indicate significance levels: p<0.05 (*), p<0.01 (**), p<0.001 (***). Error bars reflect SEMs.

Table S5. Averaged correlations (r) and effect sizes (□^{2}) between true parameters and recovered parameters for the reactive task.

	ST		SF		RT		RF	
Reactive task	r	□^{2}	r	□^{2}	r	□^{2}	r	□^{2}
□	0.55	0.43	0.69	0.89	0.46	0.27	0.57	0.48
ζ_1	0.97	17	0.97	13.48	0.98	28.38	0.97	17.18
ζ_2	0.76	1.34	0.70	0.97	0.58	0.5	0.68	0.86

ST = stable/true, SF = stable/false, RT = reversal/true, RF = reversal/false.

Table S6. Averaged correlations (r) and effect sizes (□^{2}) between true parameters and recovered parameters for the predictive task.

	ST		SF		RT		RF	
Predictive task	r	□^{2}	r	□^{2}	r	□^{2}	r	□^{2}
□	0.74	1.21	0.88	3.43	0.82	2.02	0.84	2.34
β	0.82	2.12	0.92	5.74	0.87	3.05	0.95	9.54

ST = stable/true, SF = stable/false, RT = reversal/true, RF = reversal/false.

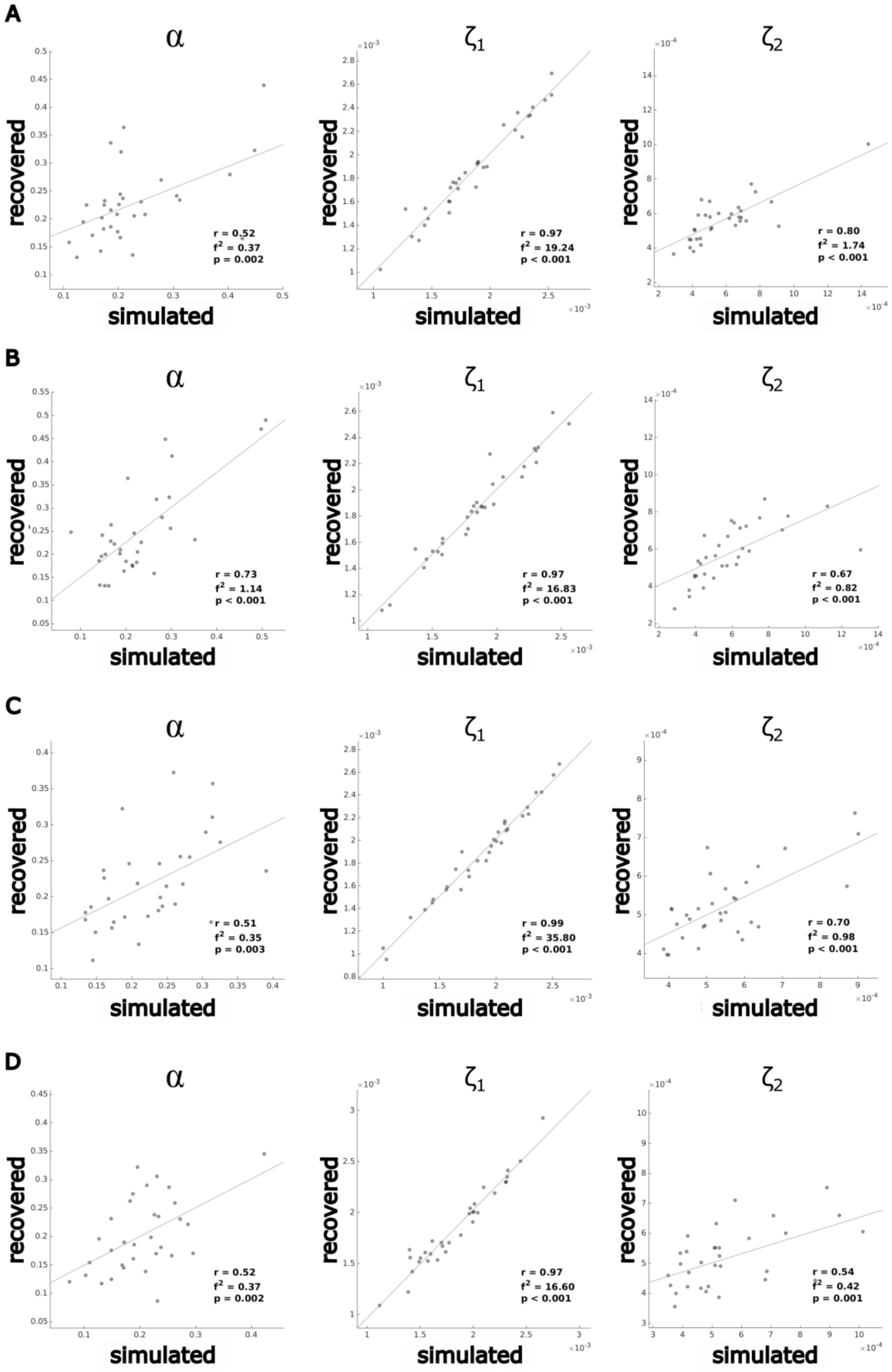

Figure S6. Parameter recovery results for the RW model parameters in the reactive task across the four conditions, shown for a randomly selected seed: **(A)** stable environment with true prior, **(B)** stable environment with false prior, **(C)** reversal environment with true prior, **(D)** reversal environment with false prior. All parameters are plotted on their native scale.

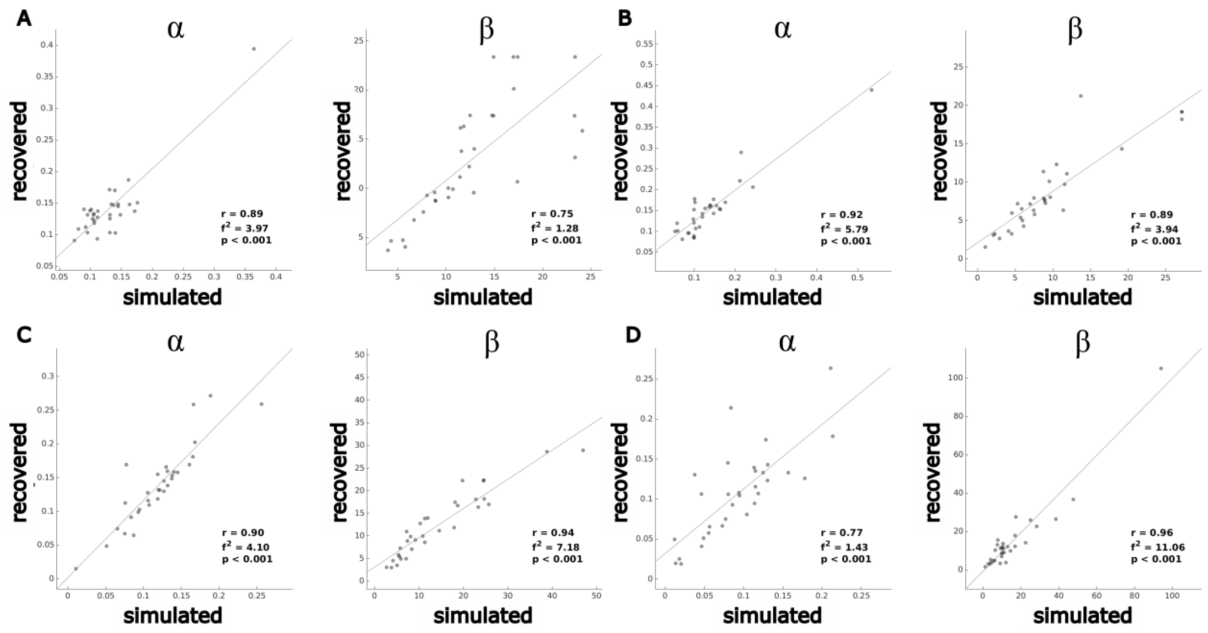

Figure S7. Parameter recovery results for the RW model parameters in the predictive task across the four conditions, shown for a randomly selected seed: **(A)** stable environment with true prior, **(B)** stable environment with false prior, **(C)** reversal environment with true prior, **(D)** reversal environment with false prior. All parameters are plotted on their native scale.

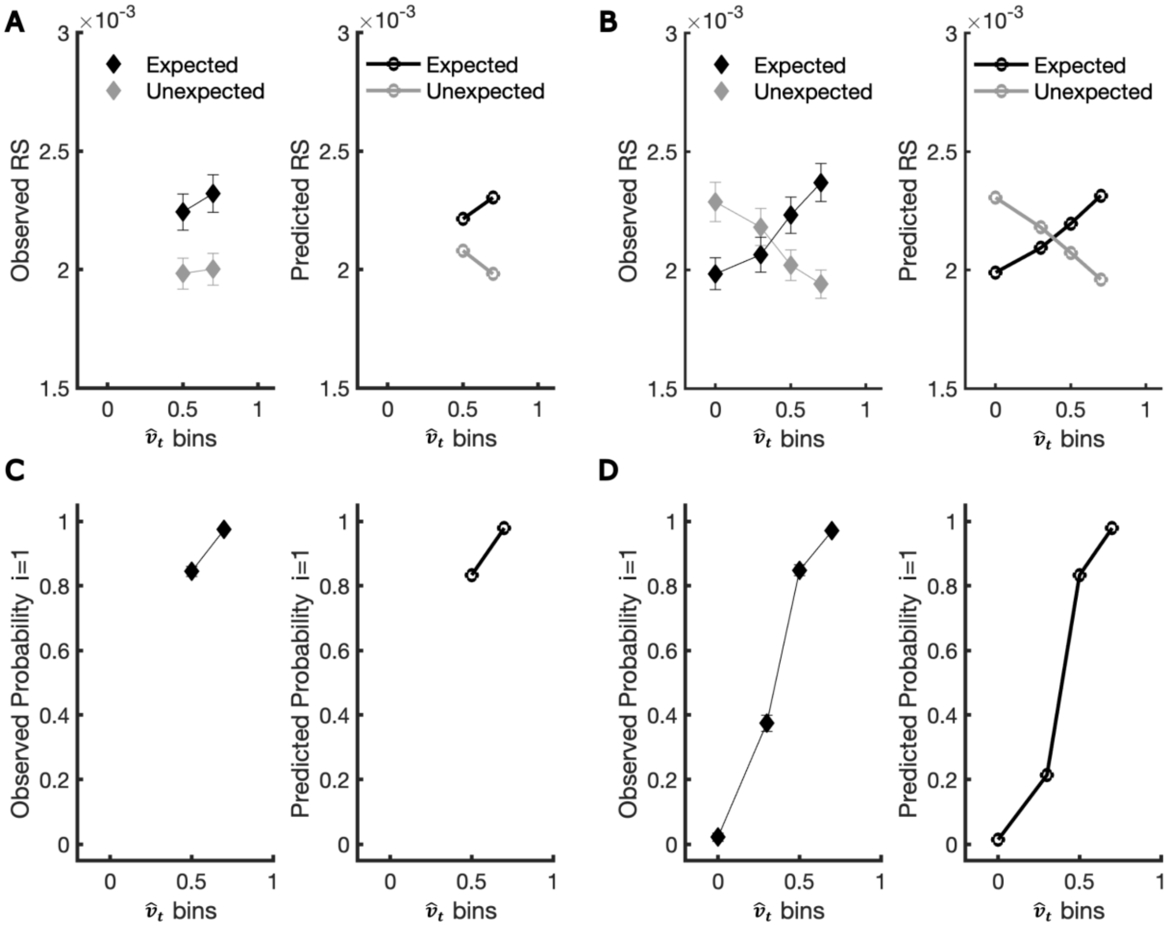

Figure S8. Additional model fit validation**.** Comparison of observed responses vs. responses predicted by the RW model based on the estimated model parameters. For each subject, we simulated responses using subject-specific perceptual and response model parameters. Both observed and simulated responses were plotted as a function of the model-derived probability $\hat{v}_t$, grouped into four bins (0 – 0.3, 0.3 – 0.5, 0.5 – 0.7, 0.7 – 1) and averaged across all subjects. The x-axis in all graphs displays the four $\hat{v}_t$ bins. In the stable environment (A, C) only the upper two bins contained data since $\hat{v}_t$ did not fall below 0.5. Top row: Predicted vs observed response speed (RS) in the reactive task, shown separately for the stable **(A)** and reversal **(B)** environment. Bottom row: Predicted vs observed choice probability (probability of choices coded as 1, i.e. matching the initial cue-target contingency) in the predictive task, also split by stable **(C)** and reversal **(D)** environment. Observed and simulated results line up closely in both tasks for all conditions, indicating a good model fit.

Part D: Eye movement results

On average, participants maintained central fixation in the reactive task for approximately 84% (□3.36% SEM) and in the predictive task for approximately 82% (□1.77% SEM) of the time between cue and target onset. Task-specific ANOVAs on the proportion of trials with eye movements to cued vs uncued sides did not reveal any significant effects, indicating that participants did not systematically shift their gaze towards the cued location in either task.

References

Mathys, C. D., Lomakina, E. I., Daunizeau, J., Iglesias, S., Brodersen, K. H., Friston, K. J., & Stephan, K. E. (2014). Uncertainty in perception and the Hierarchical Gaussian Filter. *Frontiers in Human Neuroscience*, *Volume 8-2014*. https://doi.org/10.3389/fnhum.2014.00825

Mathys, C., Daunizeau, J., Friston, K. J., & Stephan, K. E. (2011). A bayesian foundation for individual learning under uncertainty. *Frontiers in Human Neuroscience*, *5*(May), Article May. https://doi.org/10.3389/fnhum.2011.00039

Palminteri, S., Wyart, V., & Koechlin, E. (2017). The Importance of Falsification in Computational Cognitive Modeling. *Trends in Cognitive Sciences*, *21*(6), 425–433. https://doi.org/10.1016/j.tics.2017.03.011

Stephan, K. E., Penny, W. D., Daunizeau, J., Moran, R. J., & Friston, K. J. (2009). Bayesian model selection for group studies. *NeuroImage*, *46*(4), 1004–1017. https://doi.org/10.1016/j.neuroimage.2009.03.025
